# Design of antibody structure-guided epitope vaccines *in silico* to induce potent immune responses against emerging viruses

**DOI:** 10.1128/jvi.00689-25

**Published:** 2025-11-11

**Authors:** Xue-Feng Wei, Liang Zhao, Zhao Zhao, Yu-Ming Gong, Yu-Ying Zheng, Gao-Feng Cheng, Gao-Xue Wang, Bin Zhu, Wei-Guang Kong

**Affiliations:** 1 College of Animal Science and Technology, Northwest A&F University 12469, Yangling, Shaanxi, China; 2 Key Laboratory of Breeding Biotechnology and Sustainable Aquaculture, Institute of Hydrobiology, Chinese Academy of Sciences 53021 , Wuhan, China; 3 Guangxi Key Laboratory of Aquatic Biotechnology and Modern Ecological Aquaculture, Guangxi Engineering Research Center for Fishery Major Diseases Control and Efficient Healthy Breeding Industrial Technology (GERCFT), Guangxi Academy of Sciences 245477, Nanning, China; University of North Carolina at Chapel Hill, Chapel Hill, North Carolina, USA

**Keywords:** epitope identification, vaccine design, antibody screening, tilapia lake virus

## Abstract

The severe damage caused by public health emergencies in recent years indicates that epitope identification and vaccine development remain potential strategies to stop viral epidemics. Despite challenges such as comprehensive virus analysis, computer simulations and antibody-based reverse vaccinology can rapidly localize antigenic epitopes without the need to resolve protein functions, thereby supporting vaccine design. In previous studies, we developed a strategy for peptide vaccine design based on simulated epitopes for tilapia lake virus (TiLV). To further improve vaccine immunogenicity and capitalize on the significance of antibodies for epitope screening, this study presents a structural modification strategy based on antigen-antibody docking for reverse vaccinology in conjunction with single amino acid mutagenesis inducing a robust immune response in tilapia. A high-affinity scFv1 was obtained through screening from the constructed single-chain antibody fragment (scFv) library targeting TiLV. Subsequently, the key dominant epitope ^385^GYQLASEIRGTIPLSS^400^ was identified through alanine scanning and antibody binding assays based on the docking. Based on the epitope and scFv1 structure, an enhanced epitope vaccine, S390F, was obtained through single amino acid random mutation modification. Boost immunizations of tilapia elicited significant responses in IgM levels and non-specific immune enzyme activities, upregulated the expression of immune-related genes, and improved RPS of TiLV infection by approximately 25%, effectively preventing viral attack. Meanwhile, S390F activated the expression of the co-stimulatory molecule CD40, suggesting a response from immune cells. The development of peptide-based vaccines through computer-aided design effectively highlights the importance of antibodies and offers valuable insights for the control of emerging diseases.

**IMPORTANCE:**

Public health emergencies pose significant threats to both human and environmental health, and the rapid control of emerging diseases often remains challenging due to their unknown characteristics. In this context, vaccines have historically been instrumental in the fight against major diseases, with epitope vaccines emerging as a preferred approach due to their precision and efficacy. In previous studies, we developed a strategy for peptide vaccine design based on simulated epitopes for tilapia lake virus (TiLV). To further improve vaccine immunogenicity and capitalize on the significance of antibodies for epitope screening, this study presents a structural modification strategy based on antigen-antibody docking for reverse vaccinology in conjunction with single amino acid mutagenesis inducing a robust immune response in tilapia. We believe that our findings are highly relevant to the field and contribute to the ongoing efforts to combat public health threats.

## INTRODUCTION

Outbreaks of public health emergencies pose significant short-term threats to international health security and human health. Although current vaccine development technology in the life sciences fields is well-established, it still heavily relies on high pathogen analysis, which often leads to delays in critical treatment during early stages of disease outbreaks (1, 2). Bioinformatics enables high-throughput screening, facilitating rapid computational analysis and simulation. This approach conserves the resources and costs associated with traditional methods and reduces the reliance on extensive pathogenic gene and functional data for vaccine development (3). However, simple predictive methods, often used primarily for adjuvant preparation, are generally insufficient for comprehensive vaccine design (4). This highlights the need for researchers to develop an efficient vaccine strategy based on bioinformatics to address more problems and perform well in biotechnology and emergencies.

Identifying the dominant epitopes of antigens is crucial for enhancing the immunogenicity of vaccines, thereby enabling precise prevention and control of pathogens (5, 6). Epitope vaccines, derived from conserved neutralizing epitopes, simulate natural infection and stimulate an immune response through key regions of pathogens, which address shortcomings of traditional vaccines, such as poor induction of cellular immunity, high toxicity and side effects, and low neutralizing activity (7). The COVID-19 pandemic has led researchers to focus more on developing neutralizing epitopes that target the immune system, with positive effects on preventing and controlling emerging viruses (8). For instance, highly conserved epitopes that confer cross-protection were identified by targeting the neutralizing antibody CR3022 isolated from patients with severe acute respiratory syndrome coronavirus 2 (SARS-CoV-2) (9). Another study has identified 23 conventional epitopes on the surface of RBD using 380 antibodies and revealed the characteristics of antibodies that strongly neutralize viruses (10). These works indicate the potential effectiveness of identifying candidate epitope vaccines against viruses while highlighting the key role of neutralizing antibodies in epitope identification.

Reverse vaccinology, which leverages structural epitope recognition, relies on the docking of virus-targeting antibody and antigens. The analysis of dominant epitopes through binding energy calculations has significantly advanced the development of next-generation vaccines for major human and animal diseases (11–13). However, it is important to note that the binding of epitope peptides to antibodies often exhibits conformational preferences, which directly influence peptide function (14). For instance, an epitope discovery and improvement system has been developed that queries various prediction servers, integrates the results, and verifies them through molecular dynamic simulations. Two modified mutants identified by this system exhibited higher immunogenicity than natural peptides (15). Therefore, further structural modifications of epitope peptides to more closely mimic antibody binding could potentially enhance vaccine development.

The single-chain variable fragment (scFv) is the smallest functional unit with complete antigen binding activity. Due to its small size, ease of penetration, and simple preparation, scFv is recommended for use as a therapeutic antibody against diseases such as SARS-CoV-2 and HIV, as well as for identifying antigen epitopes (16–19). Phage display technology, the strategy of inserting foreign genes into phage capsid genes to display foreign proteins on their surfaces, offers significant efficiency in high-throughput antibody library screening (20, 21). During natural viral infections, antibodies produced by the body exhibit strong binding to specific antigens, resulting in persistent and protective immunity (22). Therefore, the antibody library generated by immune tissues or organs, such as the spleen and peripheral blood of infected individuals, is diverse and suitable for obtaining high-affinity neutralizing antibodies against viruses (23–25).

In recent years, sudden outbreaks of tilapia lake virus (TiLV) have been reported across several continents, including Asia, Africa, and North America. It has had a significant impact on the global tilapia aquaculture industry, with mortality rates as high as 90% (26–28). However, due to the lack of complete mechanisms as an incident virus, there are challenges in preventing and eradicating the virus. Currently, the research on the neutralizing epitopes of TiLV is still in its infancy. Only a few studies have identified some potential epitopes located on the antigen S1 through phage display technology, and these epitopes exhibit strong neutralizing activity against TiLV-positive IgM (29). Immunization experiments in Nile tilapia have indicated that S6, S8, and S10 are potential antigens, but the core antigenic epitope regions within these proteins remain uncharacterized (30). In addition, the potential antigens of TiLV have been verified by various methods, such as S2 and S4, but the identification of the neutralizing epitopes of these antigens is lacking (31, 32).

This study intends to establish a design strategy for an epitope vaccine based on targeting scFv antibody, using TiLV as a novel model. Firstly, we constructed a phage scFv library to screen for high-affinity antibodies against TiLV. Next, we used reverse vaccinology to identify the dominant antigen epitopes. The dominant epitope was optimized using computer technology methods through structural docking to enhance its binding ability to high-affinity viral antibodies. Finally, we improved the immunogenicity and anti-viral response of the epitope vaccine. As a novel strategy for epitope-based vaccine development, this study provides a theoretical basis for the development of vaccines for major human and animal diseases, as well as potential emerging infectious diseases.

## RESULTS

### Construction of TiLV scFv library and obtaining scFv1 and scFv3 through biopanning

**Fig 1:**
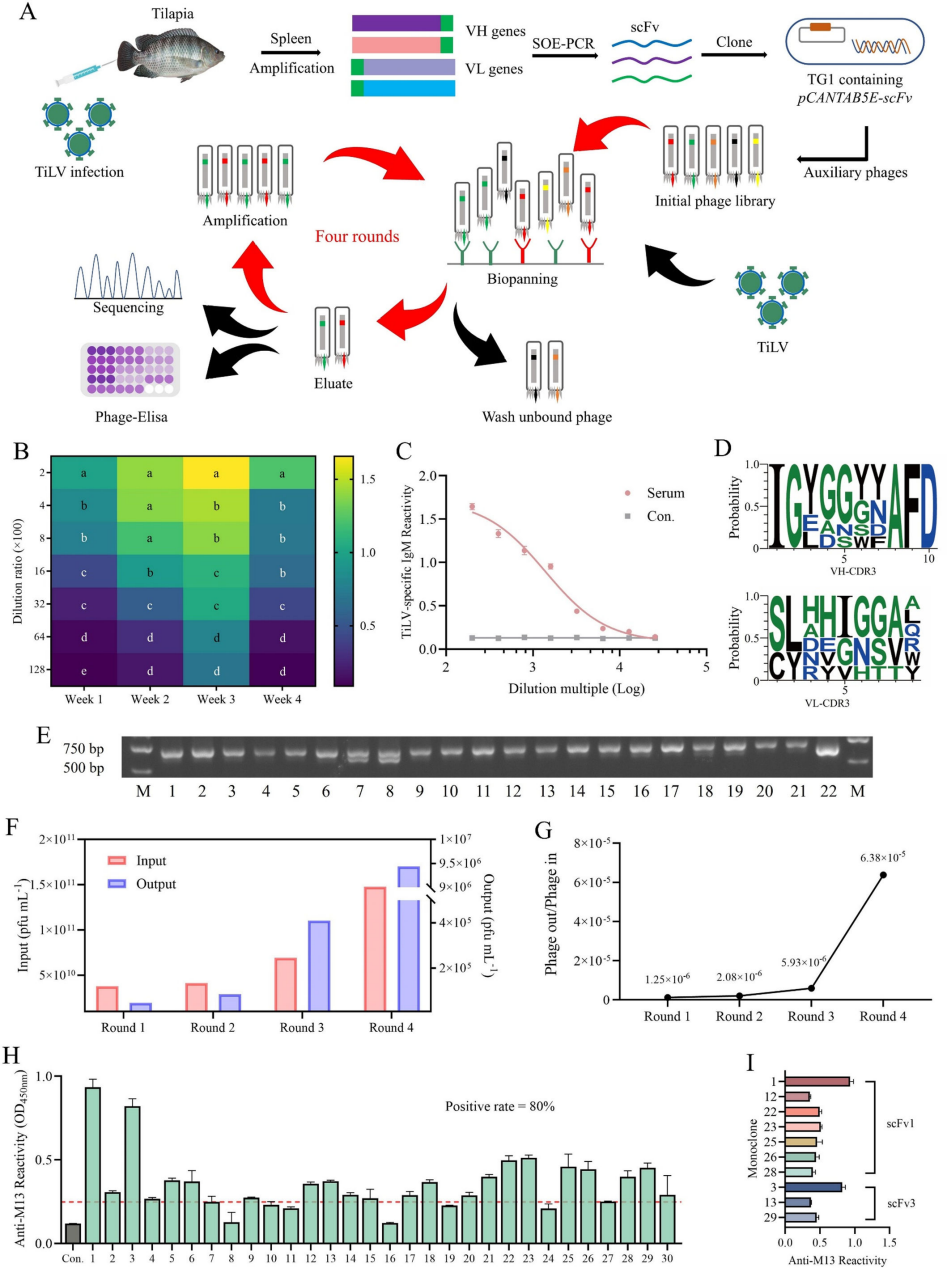
Construction of tilapia-derived antibody library and screening of TiLV-affinity antibody. (**A**) Immune antibody library construction and TiLV-affinity antibody screening strategy. Tilapias were infected with 4.0 × 10^5^ copies of the virus, and positive spleens were collected for RNA extraction. Immunoglobulin VH and VL genes were amplified using specific primers and spliced with a flexible peptide linker. The antibody library was constructed using the phage vector pCANTAB5E and strain TG1. After four rounds of bio-panning, high-affinity antibodies were sequenced and identified. (**B**) Serum samples were collected from tilapia at 1, 2, 3, and 4 weeks post-viral infection. The sera were serially diluted to different concentrations (200–12,800), and the serum antibody levels were determined using ELISA. For samples collected each week, significant differences among different dilution factors were analyzed statistically and denoted by letters, with a significance level set at *P* < 0.05. Three biological replicates were set, and the data were presented as the mean ± SD. (**C**) TiLV-specific antibody titers in tilapia serum were determined by indirect ELISA at 21 days after virus infection. (**D**) Abundance of CDR3 regions of scFv variable H and L in antibody library. The monoclonal CDR sequence in the antibody library was analyzed for richness using the Weblogo3 server after sequencing. (**E**) Recombination rate of antibody library. (**F**) The phage titers of the input and output at each round of bio-panning were determined using the plate counting method, and the enrichment factor was calculated. (**G**) Ratio of output/input in each round of bio-panning for anti-TiLV antibody library. (**H**) The high-affinity TiLV antibodies were identified by phage-ELISA. A total of 30 clones from the fourth round of elution products were randomly selected and cultured to determine affinity through phage supernatants. (**I**) The top 10 high-affinity antibodies were selected for DNA sequencing and classified according to amino acid sequence and named as scFv1 and scFv3.

The process of phage antibody library construction and specific antibody panning is illustrated in Fig. 1A. To elicit TiLV-induced immunoglobulin production in tilapia, we challenged healthy tilapia with a safe dose of virus. Spleens, a crucial immune organ and a source of immunoglobulins, were collected from surviving fish on days 7, 14, 21, and 28 post-challenges. ELISA experiments measuring antibody levels at different time points showed that the spleen, collected on day 21 after viral exposure, serves as an optimal template for constructing an antibody library (Fig. 1B). Simultaneously, the serum from day 21 also exhibits a robust binding affinity for TiLV (Fig. 1C).

The conservation of immunoglobulin framework regions 1 and 4 was analyzed using the Weblogo3 tool to design primers (Fig. S1A through D), and the variable H (VH) and L genes were specifically amplified using cDNA (Fig. S2A and B). The band sizes around 350–400 bps were consistent with expectations. Then, the scFv genes were amplified from the VH and VL genes with a flexible linker using SOE-PCR splicing. The approximately 750 bps bands represented the amplified scFv gene fragments (Fig. S2C). The correctness of the plasmid was analyzed by restriction enzyme digestion (*Not* I and *Sfi* I) after ligation of the scFv gene into the pCANTAB5E vector (Fig. S2D). The combination plasmids were transformed into TG1 competent cells to construct an antibody library with a capacity of 4.06 × 10^7^. The integrity of 22 randomly selected antibody clones was verified by agarose gel, and the recombination rate was calculated to be 100% (Fig. 1E).

Surface-coated virus was used to screen for specific antibodies, and the plate coating count determines the input and output volumes for each round of panning (Fig. 1F and Table 1). Four rounds of panning resulted in a 51-fold enrichment of TiLV-specific antibodies (Fig. 1G). In addition, the abundance of complementarity-determining regions (CDR) 3 (Fig. 1D), as well as CDR1 and CDR2 regions of the VH and VL in the screened scFv, was analyzed (Fig. S2E through H). The affinity of scFv particles from the fourth round of screening for viruses was evaluated using phage ELISA. Positive single clones accounted for 80% in the phage antibody library (Fig. 1H). Sequence analysis of the top 10 monoclonal antibodies revealed that antibodies 1, 12, 22, 23, 25, 26, and 28 were identical and named scFv1, while antibodies 3, 13, and 29 were also identical and named scFv3 (Fig. 1I).

**TABLE 1:** Phage titers of the input and output at each round of bio-panning

Round no.	Input (pfu mL^−1^)	Output (pfu mL^−1^)	Output/input
1	3.78 × 10^10^	4.74 × 10^4^	1.25 × 10^−6^
2	4.12 × 10^10^	8.57 × 10^4^	2.08 × 10^−6^
3	6.91 × 10^10^	4.10 × 10^5^	5.93 × 10^−6^
4	1.48 × 10^11^	9.44 × 10^6^	6.38 × 10^−5^
			51-fold

### scFv1 exhibits a strong affinity for TiLV

**Fig 2:**
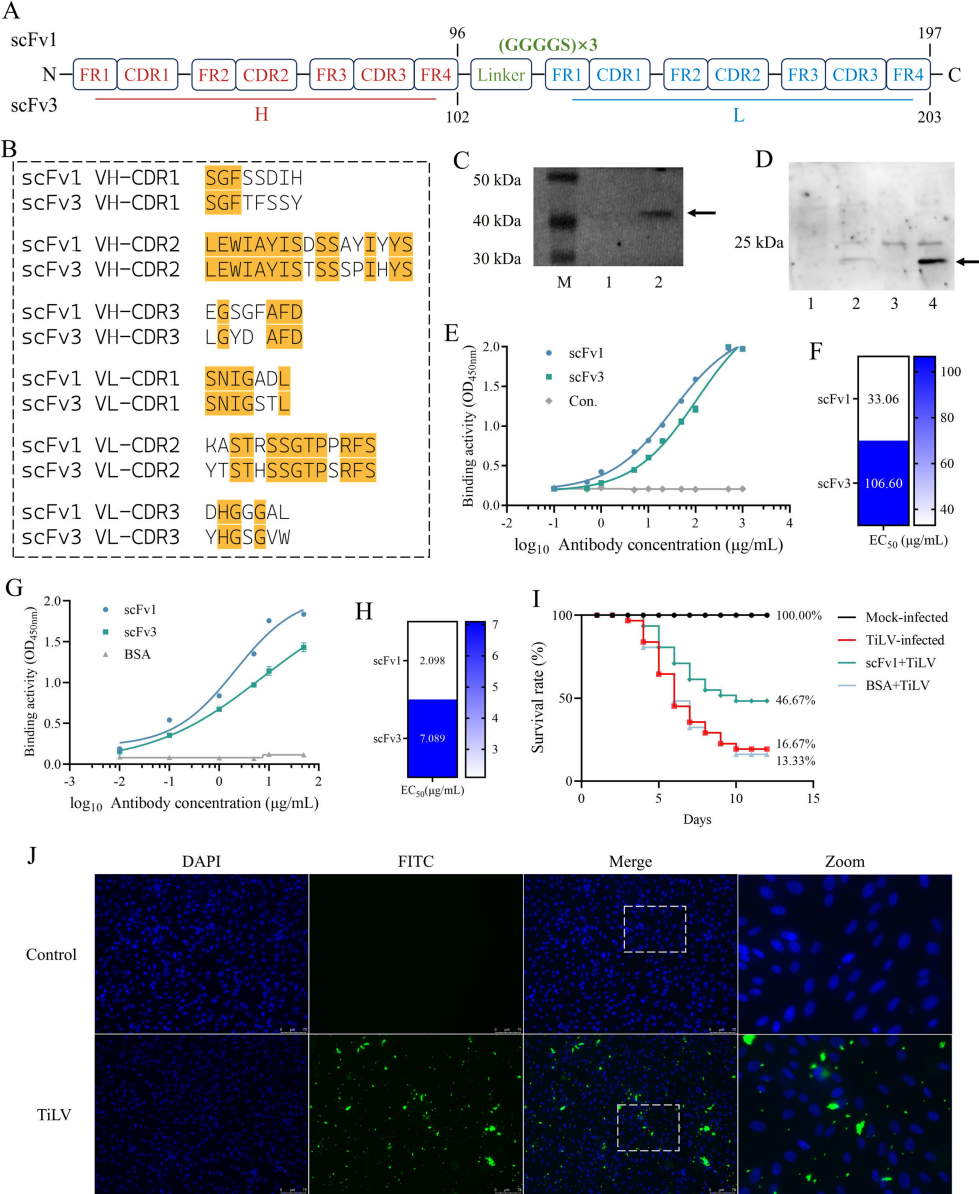
High-affinity antibody identification and bioinformatics analysis of antigen-antibody docking. (**A**) Diagrams showing the domains of scFv1 and scFv3. (**B**) Comparison of the amino acid sequences in the CDRs of scFv1 and scFv3. (**C**) The expression of antibody protein was identified by Western blot. Lane M, Marker; Lane 1, pET32a; Lane 2, scFv1. (**D**) The scFv proteins expressed in CHO cells was identified by Western blot. Lane 1: pcDNA3.1; Lane 2: scFv1; Lane 3: pcDNA3.1; Lane 4: scFv3. (**E**) The affinity of antibody scFv1 and scFv3 to TiLV at different concentrations was analyzed by indirect ELISA after prokaryotic expression. Three biological replicates were set, and the data were presented as the mean ± SD. (**F**) Analysis of the EC_50_ for the binding of scFv1 and scFv3 to TiLV. (**G**) The affinity of the scFv expressed in CHO cells to TiLV was analyzed by indirect ELISA, with the BSA protein serving as the negative control. (**H**) The EC_50_ of the scFv expressed in CHO cells that binds to TiLV was analyzed. (**I**) The protective capacity of scFv1 against TiLV infection in tilapia was evaluated via an *in vivo* protection assay. The mortality of tilapia was continuously observed for 12 days, and a viral control group and a BSA control group were set up. (**J**) Observation of the binding ability between scFv1 and TiLV through fluorescence in CIK cells.

Based on the sequencing results, we annotated the amino acid sequences of scFv1 and scFv3, including sequence length, region location, and linker location (Fig. 2A). Subsequently, a comparative analysis was conducted on the CDRs of the VH and VL chains in the amino acid sequences of scFv1 and scFv3. It was found that most of the amino acid types and positions were consistent and were marked in yellow (Fig. 2B). The same analysis was carried out for the Fr (Fig. S3F). The scFv1 and scFv3 were amplified (Fig. S3A), and the prokaryotic expression plasmids pET32a-scFv1/scFv3 (Fig. S3B and C) and the eukaryotic expression plasmids pcDNA3.1-scFv1/scFv3 (Fig. S3D) were successfully constructed. The SDS-PAGE results indicate that the size of pcDNA3.1-scFv1 and scFv3 is ~20 kDa (Fig. S3E). The Western blot experiment using the 6×His tag antibody showed that the band of the prokaryote-expressed scFv1 was approximately 40 kDa in size (Fig. 2C), and the band of the eukaryote-expressed scFv was approximately 20 kDa in size (Fig. 2D), which was consistent with the results of SDS-PAGE.

The results of the indirect ELISA indicated that scFv1 exhibited a stronger affinity to TiLV than scFv3 at the same concentration (Fig. 2E). The results of the EC_50_ analysis indicate that the concentration of scFv1 required to bind to half of the TiLV is 33.06 µg/mL, which is 3.22-fold lower than that of scFv3 (Fig. 2F). Similarly, the scFv proteins obtained through the CHO expression system also demonstrated that scFv1 had a stronger ability to bind to TiLV compared to scFv3, and the BSA control group did not exhibit binding to TiLV (Fig. 2G). The EC_50_ values were 2.098 µg/mL and 7.089 µg/mL, respectively (Fig. 2H). In addition, the *in vivo* protection studies in tilapia showed that scFv1 could alleviate the damage of tilapia infected with TiLV and increase the survival rate by nearly 30%. The BSA control group did not improve the mortality rate of tilapia infected with TiLV, and the tilapia without TiLV injection did not die during the observation period (Fig. 2I). The results of immunofluorescence showed that a large amount of green fluorescence was observed on CIK cells infected with TiLV, indicating that the scFv1 protein had bound to TiLV (Fig. 2J).

### Antigen-antibody docking and mutation analysis of binding energy

**Fig 3:**
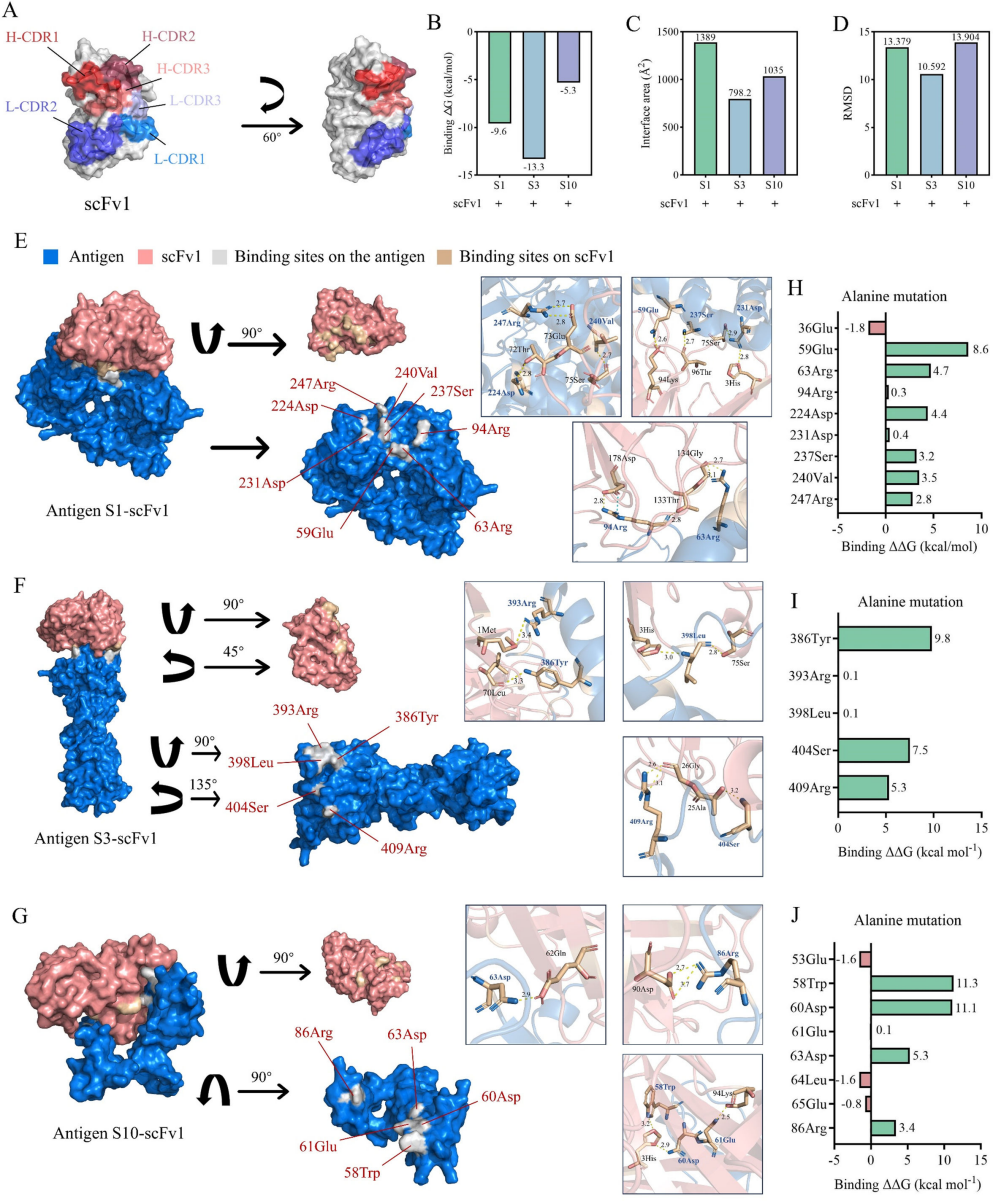
Analysis of antigen-antibody docking alanine mutation and the three-dimensional interface. (**A**) The three-dimensional structure models were predicted by Alaphfold2, and the CDRs of scFv1 were identified using PyMOL based on the amino acid sequences, respectively. (**B**) Analysis of the binding energy of the complexes of scFv1 with TiLV antigens S1, S3, and S10, respectively. (**C**) Analysis of the interaction area of the complexes of scFv1 with TiLV antigens S1, S3, and S10, respectively. (**D**) Analysis of the RMSD of the complexes of scFv1 with TiLV antigens S1, S3, and S10, respectively. (**E**) Docking model for scFv1 and antigen S1, with antigens colored marine, scFv1 colored salmon, and binding amino acids colored wheat. The hydrogen bonds and key amino acids in the three-dimensional docking interface between antibody scFv1 and S1 were mapped using PyMOL. The alanine mutations were introduced to the amino acids that were binding to the antigen S1, and the binding energy to scFv1 was then recalculated. (**F**) Docking model for scFv1 and antigen S3. (**G**) Docking model for scFv1 and antigen S10. (**H**) The alanine mutations were introduced to the amino acids that were binding to the antigen S1, and the binding energy to scFv1 was then recalculated. (**I**) Antigen 3. (**J**) Antigen 10.

AlphaFold2 provided structural models of scFv1 and scFv3 based on their amino acid sequences, which were then presented in PyMOL. Meanwhile, the positions of all CDRs of the VH and VL chains were marked with different colors (Fig. 3A; Fig. S3G), and amino acid information is shown in Table 2. Subsequently, the amino acids of scFv1 and the quality of its model were analyzed. Objective evaluations using the ProSA tool were conducted on the scFv models. The Z-score of the scFv1 model is −6.77, as shown in Fig. S4A. The quality of the local model is demonstrated by plotting the correlation between energy and the position of the amino acid sequence (Fig. S4B). Furthermore, scFv1 contained more hydrophilic (negatively charged) than hydrophobic (positively charged) amino acids, indicating a stronger affinity for hydrophilicity (Fig. S4C). Electrostatic potential maps were produced using PyMOL to visualize the charge distribution of the molecular residues (Fig. S4D). The VERIFY 3D result displayed in Fig. S4E illustrates the correlation between the atomic model (3D) and the corresponding amino acid sequence (1D), with an average 3D-1D score of ≥ 0.2 across all residues, indicating that the models were of high quality.

**TABLE 2:** Amino acid sequence information for scFv1 and scFv3

Name	Amino acid sequence
scFv1	MSHRLTCAYSGFSSDIHAVWVRQAAGKGLEWIAYISDSSAYIYYSQSVRGRFT ISRDNSRKQVYLQMNSLTTEDSAVYYCTREGSGFAFDYWGKGTGGGGSGGG GSGGGGSVTISATGSSNIGADLSWYLQKTGETPKLLIYKASTRSSGTPPRFSGS TSGSQYTLTISGVQAEDAGDYYCLGDHGGGALTFGGGTK
scFv3	MVKRPGESHRLTCTTSGFTFSSYRMVWVRQAPGKGLEWIAYISTSSSPIHYSQ SVQGRFTISRDDSSSKVYLQMNTLKTEDTAVYYCARLGYDAFDYWGKGTGG GGSGGGGSGGGGSVTISATGSSNIGSTLSWYLQKPGQPPKLLIYYTSTHSSGTP SRFSGSRSGSQYTLTISGFQPEDAGDYYCLGYHGSGVWTFGGGTK

The Cluspro server was utilized for antigen-antibody docking with default parameters. Amino acid binding sites, binding modes, hydrogen bond lengths, and hydrophobic amino acid sites from the docking results of S1-S10 of TiLV to scFv1 were analyzed using Ligplot. Based on the docking results, a total of 89 potential binding sites were identified for 10 antigen proteins (Table S1). Furthermore, the PDBe tool was employed to analyze the binding energies and interface areas (Fig. 3B and C). RMSDs of scFvs and 10 antigens were analyzed using PyMOL (Fig. 3D). The interaction interfaces of scFv1 with S1, S3, and S10 antigens are illustrated in graphical form. The formation of hydrogen bonds and the length of bonds are indicated by yellow dotted lines, while magenta dotted lines represent the formation of salt bridges, which are better understood (Fig. 3E through G). And the results of other antigens were shown in Fig. S5.

Alanine mutations were performed on each potential site to analyze the effect on antigen-antibody docking. The position of the potential binding sites on the antigens and the binding energy results of its mutation were reanalyzed (Fig. 3H through J; Fig. S6). The findings indicate that 54 potential binding sites exhibited reduced binding ability to scFv1 following mutation (i.e.. G less than 0), suggesting that these sites may contribute to docking.

### Validate ^385^GYQLASEIRGTIPLSS^400^ as a potential epitope peptide both *in vitro* and *in vivo*

The region where the positive binding site is located may represent a potential linear epitope capable of eliciting a more robust immune response *in vivo*. Therefore, three antigenic epitopes, each consisting of 12–20 amino acids, were identified on the S1, S3, and S10 antigens to evaluate their binding capacity to antibodies. All of these antigenic epitopes carried at least three different positive binding sites (Table 3).

**TABLE 3:** Information on S1, S3, and S10 dominant epitopes and their alanine mutant strains

Antigen	Domain	Sequences	Key amino acids
S1-WT	231–250	DAIGMGSIGVMLAYMVRRKC	237S, 240V, 247R
S1-Mut		DAIGMGAIGAMLAYMVARKC	
S3-WT	385–400	GYQLASEIRGTIPLSS	386Y, 393R, 398L
S3-Mut		GAQLASEIAGTIPASS	
S10-WT	55–66	NIEWGDEVDLEM	58W, 60D, 61E, 63D
S10-Mut		NIEAGAAVALEM	

**Fig 4:**
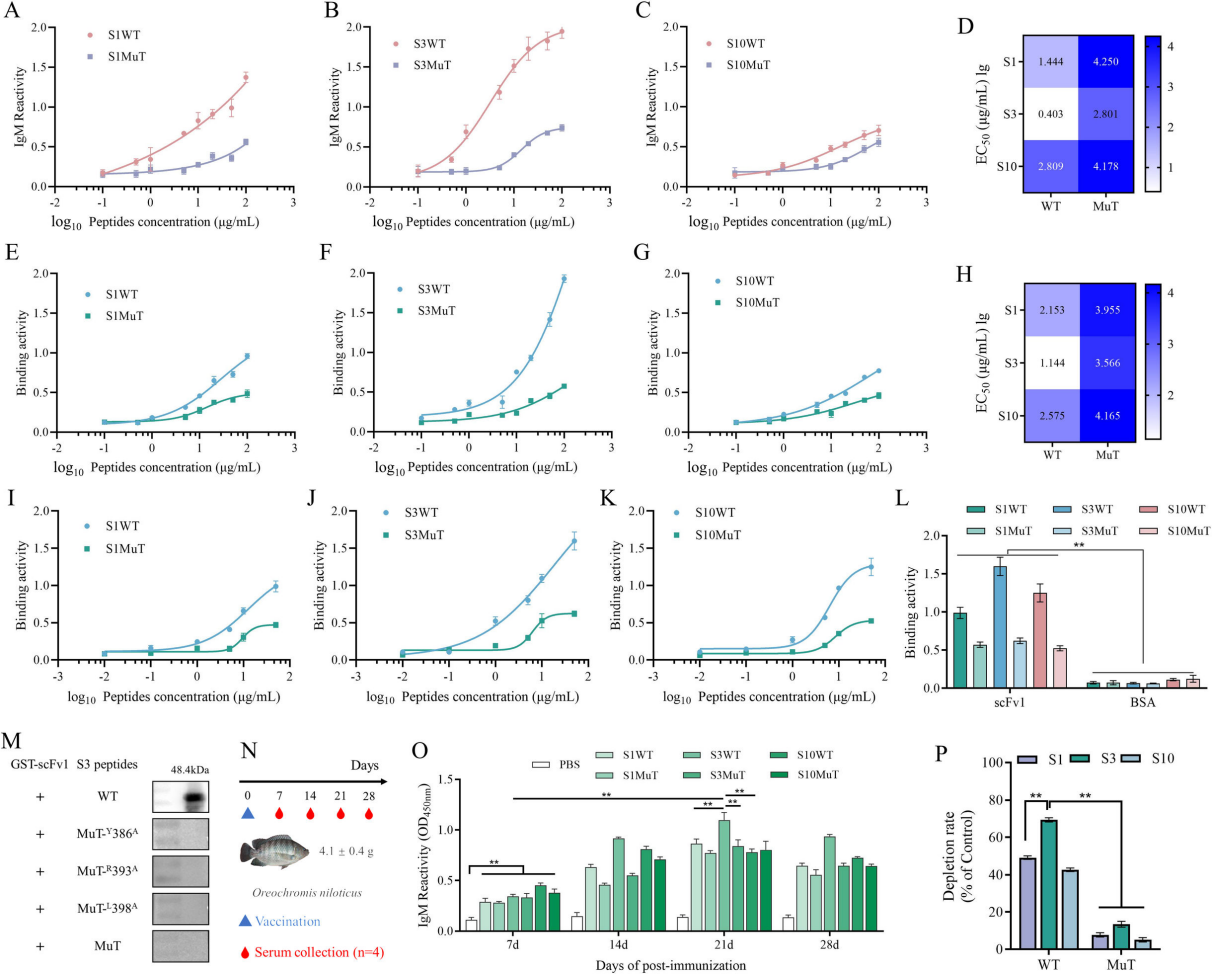
Identification of the dominant S3 epitope. (**A**) The affinity of epitope peptides (S1WT and S1MuT) to TiLV-positive serum (1:200) at various concentrations was determined by indirect ELISA. The scFv1 was obtained using the prokaryotic expression system of *E. coli* Bl21 with a concentration of 50 µg/mL. The epitope peptides were synthesized using the solid phase method. Three biological replicates were set, and the data were presented as the mean ± SD. (**B**) S3WT and S3MuT. (**C**) S10WT and S10MuT. (**D**) Analysis of the EC_50_ of epitope peptides in binding to TiLV-positive IgM. (**E**) The binding capacity of epitope peptides to antibody scFv1 (50 µg/mL) at various concentrations was determined by indirect ELISA. After prokaryotic expression, the scFv1 was freeze-dried, dissolved in PBS buffer, and its concentration was analyzed by the BCA method. (**F**) S3WT and S3MuT. (**G**) S10WT and S10MuT. (**H**) Analysis of the EC_50_ of epitope peptides in binding to scFv1. (**I**) After the eukaryotic expression of scFv1 through the CHO expression system, the affinity of scFv1 for the epitope peptides (S1WT and S1MuT) was analyzed by indirect ELISA. The concentration of the epitope peptides was 50–0.01 μg/mL, and the concentration of the scFv1 protein was 50 µg/mL. Three replicates were set up. (**J**) S3WT and S3 MuT. (**K**) S10WT and S10MuT. (**L**) Using 50 µg/mL of BSA protein as a negative control, the binding ability to the epitope peptides at the highest concentration used in the experiment (50 µg/mL) and the mutants was analyzed. Three replicates were performed. **P < 0.01. (**M**) Identification of the binding ability between the S3 epitope peptide and its mutant peptides with scFv1 by Western blot. scFv1 is conjugated with a GST tag. (**N**) Diagram of epitope peptides immunization and sample collection. The immunogenicity of the epitope peptide was analyzed by immunizing tilapia. (**O**) The serum antibody titers of tilapia were determined by direct ELISA at 7, 14, 21, and 28 days after immunization. Serum was coated directly overnight in a 96-well plate and detected with mouse anti-tilapia IgM mAb. Four biological replicates were set, and the data were presented as the mean ± SD. **P < 0.01. (**P**) Detection of the TiLV-positive antibody depletion rate of the S1, S3, and S10 epitope peptides and their mutant peptides by ELISA. The data were presented as the mean ± SD. **P < 0.01.

In this study, we utilized anti-TiLV-positive serum and scFv1 protein to assess the binding capacity of wild-type antigenic peptides and their mutants to antibodies. The ELISA results indicated that the mutants of all three antigenic peptides had a reduced binding capacity to TiLV-positive antibodies, suggesting that the residues on the antigenic peptides made a significant contribution to antigen-antibody binding (Fig. 4A through C). The binding with scFv showed consistent results (Fig. 4E through G). Meanwhile, the S3WT peptide demonstrated strong binding, indicating that this specific region of S3 has a high immunogenic potential. In addition, the EC_50_ analysis of the epitope peptides binding to TiLV-positive antibodies and scFv1 also indicated the high binding activity of the S3 epitope peptide (Fig. 4D and H). Similarly, the affinity between the scFv1 obtained from the CHO expression system and the epitope peptides was analyzed by indirect ELISA, yielding similar results. The epitope peptides exhibit good affinity with scFv1, and the mutant strains show a decrease in affinity to varying degrees (Fig. 4I through K). Meanwhile, the BSA protein was used as a control to analyze the non-specific binding of the epitope peptides. The results indicate that the 50 µg/mL BSA protein cannot bind to the epitope peptides at the highest concentration used in the experiment, which further enhances the rigor of the study (Fig. 4L).

In addition, the Western blot method was used to verify the S3 peptide and its mutants. The scFv1 conjugated with a GST tag was able to bind to the S3 peptide, and a 48.4 kDa protein was recognized by the 6×His tag antibody. However, the alanine-mutated peptide did not show the protein, indicating that the alanine mutations at positions 386, 393, and 398 could not bind to scFv1 (Fig. 4M).

To evaluate the immunogenicity of the antigenic epitopes, we further determined the serum antibody titer in the vaccinated fish following the experimental procedure (Fig. 4N). The immunological assays demonstrated that the S1WT, S3WT, and S10WT antigenic peptides all elicited robust immune responses in the organism, with S3WT exhibiting the highest immunogenicity, which was consistent with the results of the affinity validation. In contrast, the mutant strains (S1MuT, S3MuT, S10MuT) showed a lower antibody stimulation level compared to the wild-type antigenic peptide and induced weaker immune responses. This indicated that mutations in pivotal binding amino acids may result in the loss or downregulation of immunogenicity in antigenic peptides, potentially serving as a guide for the design of epitope vaccines for TiLV (Fig. 4O). The TiLV-positive term depletion experiments on the S1, S3, and S10 epitope peptides showed that the S3 epitope peptide could significantly deplete TiLV-positive antibodies, with a depletion rate approaching 70%. In contrast, the depletion rates of the mutant peptides were relatively low (Fig. 4P). The results suggest that the ^385^GYQLASEIRGTIPLSS^400^ fragment of S3 displays significant antigenicity that is exploitable for further epitope mining and vaccine design.

### Analysis of single amino acid mutation and design of the S390F epitope peptide

**Fig 5:**
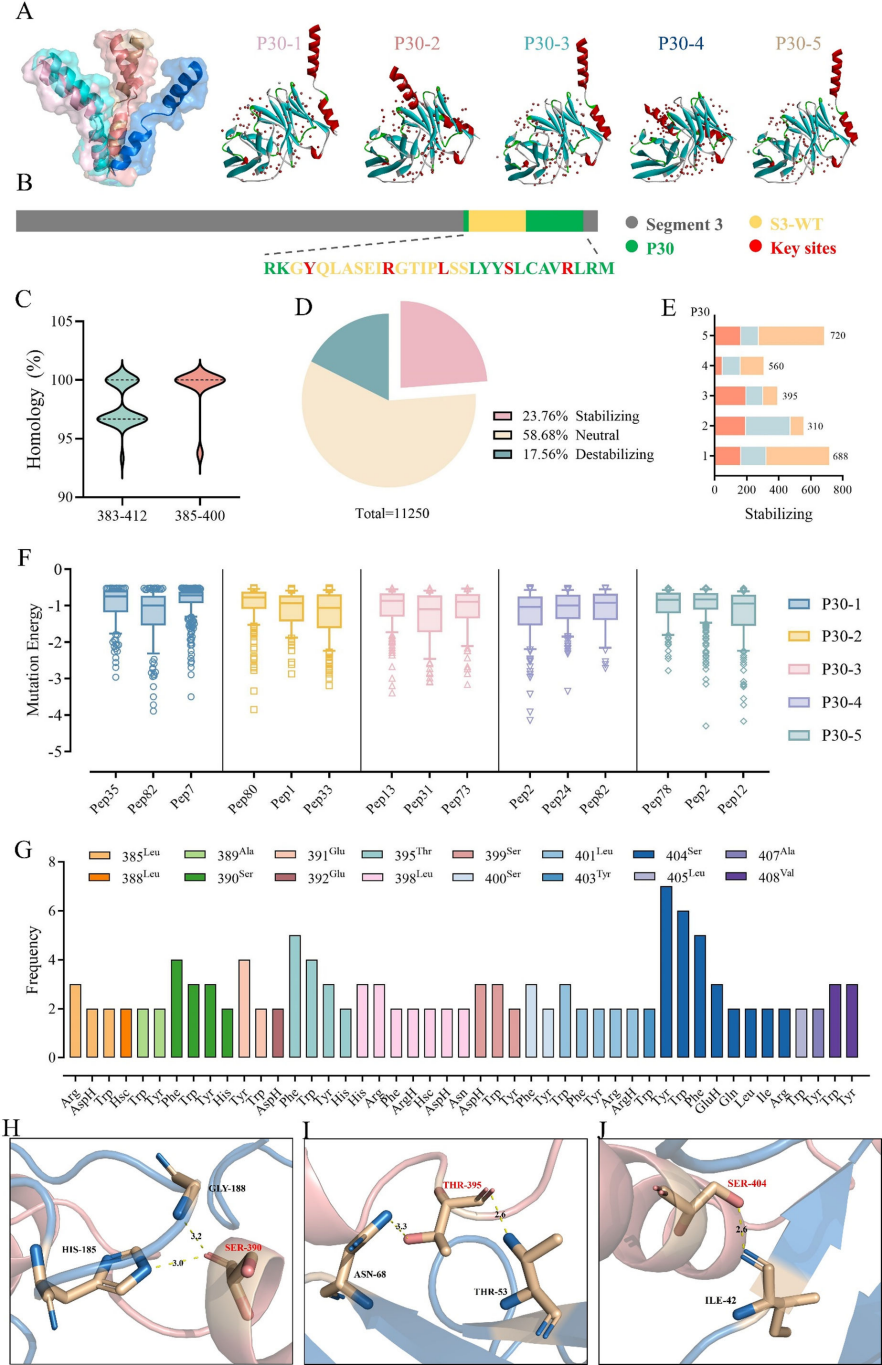
Epitope vaccine design based on P30 peptide and protein interaction verification. (**A**) A total of 5 models of P30 were predicted by Alaphfold2 and named P30-1, P30-2, P30-3, P30-4, and P30-5, respectively. The docking posture of the P30 model with antibody scFv1 was analyzed, and the P30 in red and the antibody scFv1 in cyan. The red dot represents the central site of the P30 peptide docking with the antibody in different postures. (**B**) The diagram illustrates the position relationship and sequence information of S3-WT and P30 on antigen S3. P30 is highlighted in green, S3-WT in yellow, and the remaining portions are in gray. Key amino acid residues are marked in red. (**C**) The violin plot showed the homology of 383–412 and 385–400 sites of different TiLV strain S3. (**D**) Proportions of stable, neutral, and unstable structures among all single amino acid mutation results of the P30 peptide. (**E**) The number of mutations resulting in stable structures after single amino acid random mutations in different poses of the five models of the P30 peptide. (**F**) Changes in mutation energy after single amino acid random mutations in 15 docking poses. (**G**) Each site of the P30 peptide underwent 25 single-point amino acid mutations, and the binding energy to the scFv1 was recalculated. The types of amino acid mutations with binding energy less than −2 on each residue were displayed from 750 results, and the frequency of occurrence was counted. Mutations with a frequency of 1 are not shown. (**H**) The three-dimensional docking of 390Ser in P30 peptide with antibody scFv1 was drawn by PyMOL, and this site performed well in single-point amino acid mutation analysis. (**I**) 395Thr. (**J**) 404Ser.

The S3-WT antigenic peptide performed well in antibody binding and immune response induction and was subsequently modified to enhance its immunogenicity. Peptide chain elongation has been identified as an effective means of enhancing both structural stability and epitope activity (33). To this end, we have identified an epitope based on S3-WT peptide, named P30, covering sites 383–412 on the S3 antigen, including all the crucial residue sites required for the docking of the S3 protein with scFv1. AlphaFold2 predicted five structural models for the P30 peptide. P30 models were subjected to Z-dock protein docking with scFv1. The P30 peptides were spatially translated and rotated relative to the scFv1 model to explore all plausible binding modes. Dots indicating the center of the pose of P30 peptide were generated. The red represents higher binding energy, while the blue represents lower binding energy scores (Fig. 5A). The S3 dominant epitope and P30 epitope positions were distinguished by different colors, and the key docking sites were highlighted in red (Fig. 5B). Additionally, Mega6 software and Weblogo3 tool were utilized to analyze the conservation of S3 sequences of all identified TiLV provided by NCBI (Fig. S7). The results showed that the 385–400 epitope homology was above 93.75% and was consistent with 29 sequences. The 383–412 epitope was also highly conserved, with over 93.33% homology (Fig. 5C).

The three most distinct binding poses out of 2,000 were chosen for single-point amino acid mutations in order to investigate patterns of higher binding capacity. For each pose, the P30 peptide was subjected to 25 single-point mutations of amino acids, resulting in a total of 750 outputs. The stability of the mutant structures was analyzed from a total of 11,250 output results (Table S5). The majority of the results were neutral, and 23.76% of the output results showed more stable structures (Fig. 5D). The number of stable structures after mutation in three different docking poses was also analyzed in five different P30 models (Fig. 5E). Meanwhile, a box plot presented the mutation energy of the stable structures under 15 docking poses. The overall mutation energy was around −1. The lower the value, the more stable the structure (Fig. 5F). In addition, we analyzed the number of neutral and unstable structures after mutation in three different docking poses within five P30 models (Fig. S8A and B). The results were consistent with those in Fig. 5D. We also analyzed the van der Waals (VDW) forces and electrostatic potential of the stable structures across 15 docking poses (Fig. S8C and D). These values generally changed in line with the mutation energy, indicating that VDW and electrostatic potential can also serve as guiding indicators for the stability of mutant structures.

All single-point mutations were screened and considered valid if two criteria were met: (1) The P30 peptide docked with scFv1 and produced a stable structure, and (2) the mutation binding energy was lower than −2. The study revealed that the S3 antigen had the highest incidence of serine (Ser) to tyrosine (Tyr) mutations at position 404, followed by Ser to tryptophan (Trp), serine to phenylalanine (Phe) mutations at position 404, threonine (Thr) to phenylalanine, threonine to tryptophan mutations at position 395, serine to phenylalanine mutations at position 390, and glutamic acid (Glu) to Tyr mutations at position 491 (Fig. 5G). The three-dimensional docking of three common mutant amino acids (S390, T395, and S404) was analyzed in PyMOL to improve understanding (Fig. 5H through J). Following the design of mutants that can provide stronger binding according to the docking structure (Table 4), the next step was to assess the effect of these mutant peptides on positive serum affinity and antibody neutralization.

**TABLE 4:** P30-based epitope vaccine amino acid sequence

Name	Sequence
P30	RKGYQLASEIRGTIPLSSLYYSLCAVRLRM
S390F	RKGYQLAFEIRGTIPLSSLYYSLCAVRLRM
T395F	RKGYQLASEIRGFIPLSSLYYSLCAVRLRM
S404Y	RKGYQLASEIRGTIPLSSLYYYLCAVRLRM
G394K	RKGYQLASEIRKTIPLSSLYYSLCAVRLRM

In addition, the types of mutation frequencies under the two mutation energy thresholds (< −2 kcal/mol and <−1.5 kcal/mol) were analyzed, and the top 10 were ranked. The results show that mutation types under the two thresholds were highly similar, and the three mutation types selected for further analysis in this study ranked among the top ones (Table S2).

### The S390F epitope peptide has a strong interaction with scFv1

**Fig 6:**
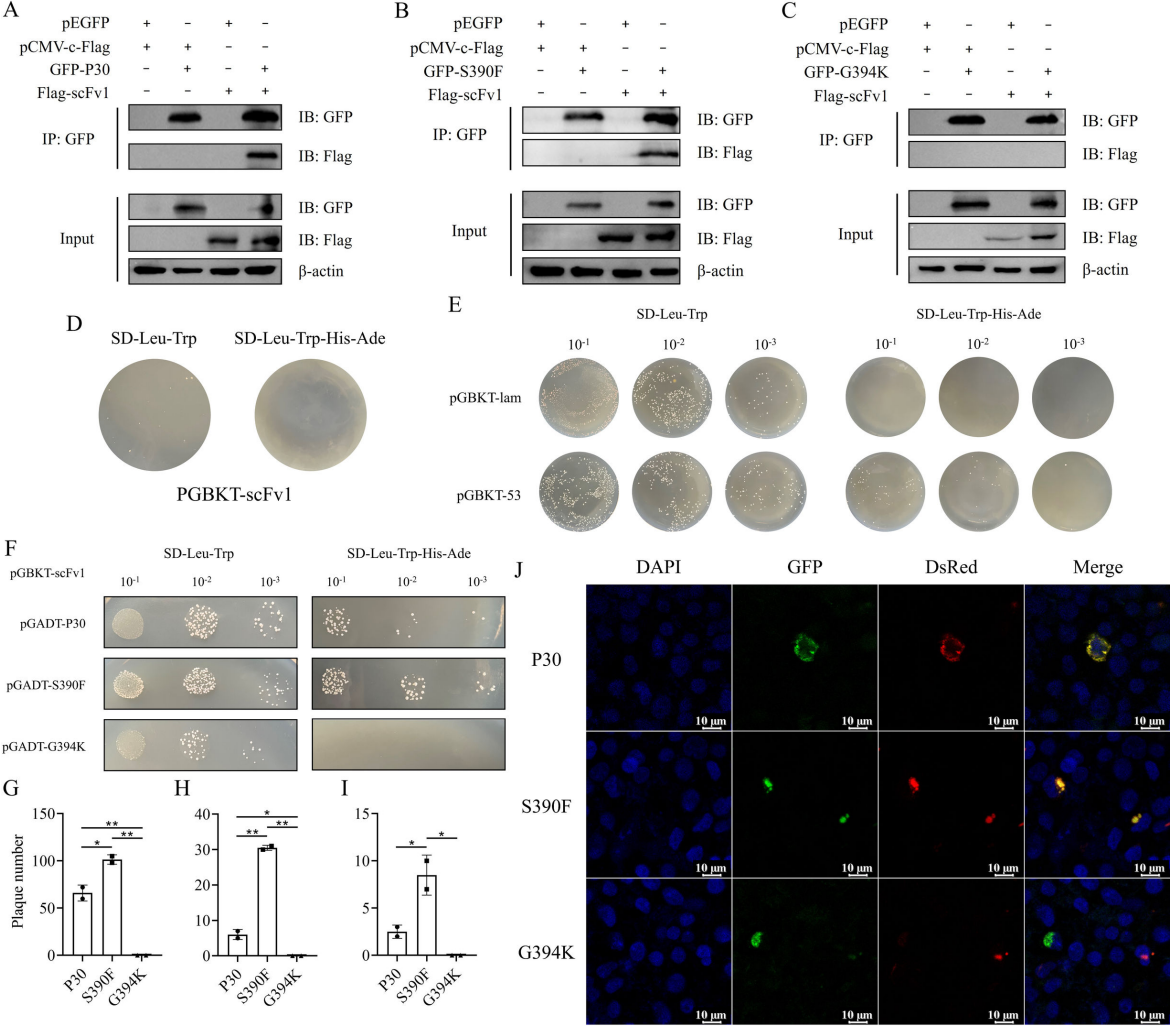
Analysis of protein interactions of epitope peptides with scFv1. (**A**) Analysis of protein interaction between P30 peptide and scFv1. Plasmids pEGFP-P30 and pCMV-Flag-scFv1 were constructed and co-transfected into HEK293T cells. After 48 h, the proteins were collected and incubated with magnetic beads treated with mouse anti-GFP mAb. The samples were eluted with SDS-PAGE loading buffer and verified by Western blot. Plasmids pEGFP and pCMV empty vector were used as controls. (**B**) Analysis of protein interaction between S390F peptide and scFv1. (**C**) Analysis of protein interaction between G394K peptide and scFv1. (**D**) The self-activation verification of the bait plasmid PGBKT-scFv1 on SD-deficient medium. (**E**) Negative and positive controls in yeast two-hybrid assays. The pGADT-T plasmid was co-transformed with pGBKT-lam and pGBKT-53 plasmids in Y_2_Hgold competent cells as negative and positive controls, respectively. (**F**) The protein interactions between scFv1 and the P30, S390F, and G394K peptides were analyzed by yeast two-hybrid assay. After dilution, the bait plasmid pGBKT-scFv1 was co-transformed with the pGADT-P30, S390F, and G394K plasmids in Y_2_Hgold competent cells and cultured in SD-Leu-Trp and SD-Leu-Trp-His-Ade deficient medium for 48–96 h. (**G**) After a 10-fold dilution, the number of plaques on SD-LTHA deficient medium of competent cells co-transformed with pGADT-P30, S390F, and G394K with pGBKT-scFv1 plasmids was counted. *P < 0.05. **P < 0.01. (**H**) After 100-fold dilution, the number of plaques on SD-LTHA deficient medium. *P < 0.05. **P < 0.01. (**I**) After 1,000-fold dilution, the number of plaques on SD-LTHA deficient medium. *P < 0.05. **P < 0.01. (**J**) The co-localization of P30, S390F, and G394K peptides with scFv1 was observed using laser confocal microscopy. To achieve this, pEGFP-P30, S390F, and G394K were co-transfected with pCMV-DsRed-scFv1 into HEK293T cells and cultured for 48 h. The fluorescence expression of the proteins was observed and analyzed under a 63× confocal microscope after DAPI staining with an anti-fluorescence quencher.

To further investigate the relationship between the mutant peptides and the antibody, we performed a protein interaction assay using the S390F mutant peptides, which showed good affinity improvement; the non-mutated P30 peptide; and the G394K mutant peptides, which showed negative improvement. The results showed that both the P30 and S390F peptides were able to bind to the antibody and were eluted from magnetic beads that were incubated with the GFP-tagged antibody (Fig. 6A and B). However, G394K was negative in the Co-IP reaction with the scFv1, and the IP sample was not recognized by the Flag-tag antibody (Fig. 6C). To rule out the interference of large tagged proteins, the interactions between P30, S390F, G394K, and scFv1 proteins were also analyzed using the His-tag system. The results were consistent with those of the GFP expression system. P30 and S390F showed interactions with scFv1, while G394K did not (Fig. S9A through C). Consistent conclusions were further drawn using Western blot analysis, and no significant bands were observed for the BSA protein (Fig. S9D).

A yeast two-hybrid experiment was then performed using the antibody as the bait protein and the mutant peptides as the target proteins. The observation that the bait plasmid pGBKT-scFv1 grew in Leu/Trp-deficient (LT) SD medium and failed to grow in Leu/Trp/His/Ade-deficient (LTHA) SD medium indicated that the scFv1 bait plasmid was not self-activated and was available for subsequent interaction experiments (Fig. 6D). In addition, the bait plasmids pGBKT-lam and pGBKT-53 were co-transformed with pGADT-T7 in yeast Y2Hgold-competent cells for negative and positive experiments, respectively. The results were as expected (Fig. 6E). The yeast two-hybrid experiments of P30 and its mutant peptides showed that P30 and S390F peptide groups exhibited plaque growth on LTHA-deficient SD medium after 96 h of co-transformation of yeast cells, under conditions where LT-deficient SD medium was available for cultivation, and the results varied significantly with the inoculation amount. However, the G394K peptide did not exhibit any growth (Fig. 6F). We measured that plaque on the LTHA-deficient SD medium and found that the Phe-Ser mutation at position 390 significantly increased binding to the antibody (Fig. 6G through I). Additionally, the co-localization in cells was observed to gain a better understanding of the interaction between P30 and mutant peptides and antibody. The peptide carrying the GPF tag appeared green, while the antibody carrying the DsRed tag appeared red. The nucleus was stained blue with DAPI. The results demonstrated that P30 and S390F peptides co-localized with the antibody, resulting in superimposed colors. However, the G394K peptide failed to bind to the antibody (Fig. 6J).

### S390F epitope peptide exhibits a high binding ability to antibodies

**Fig 7:**
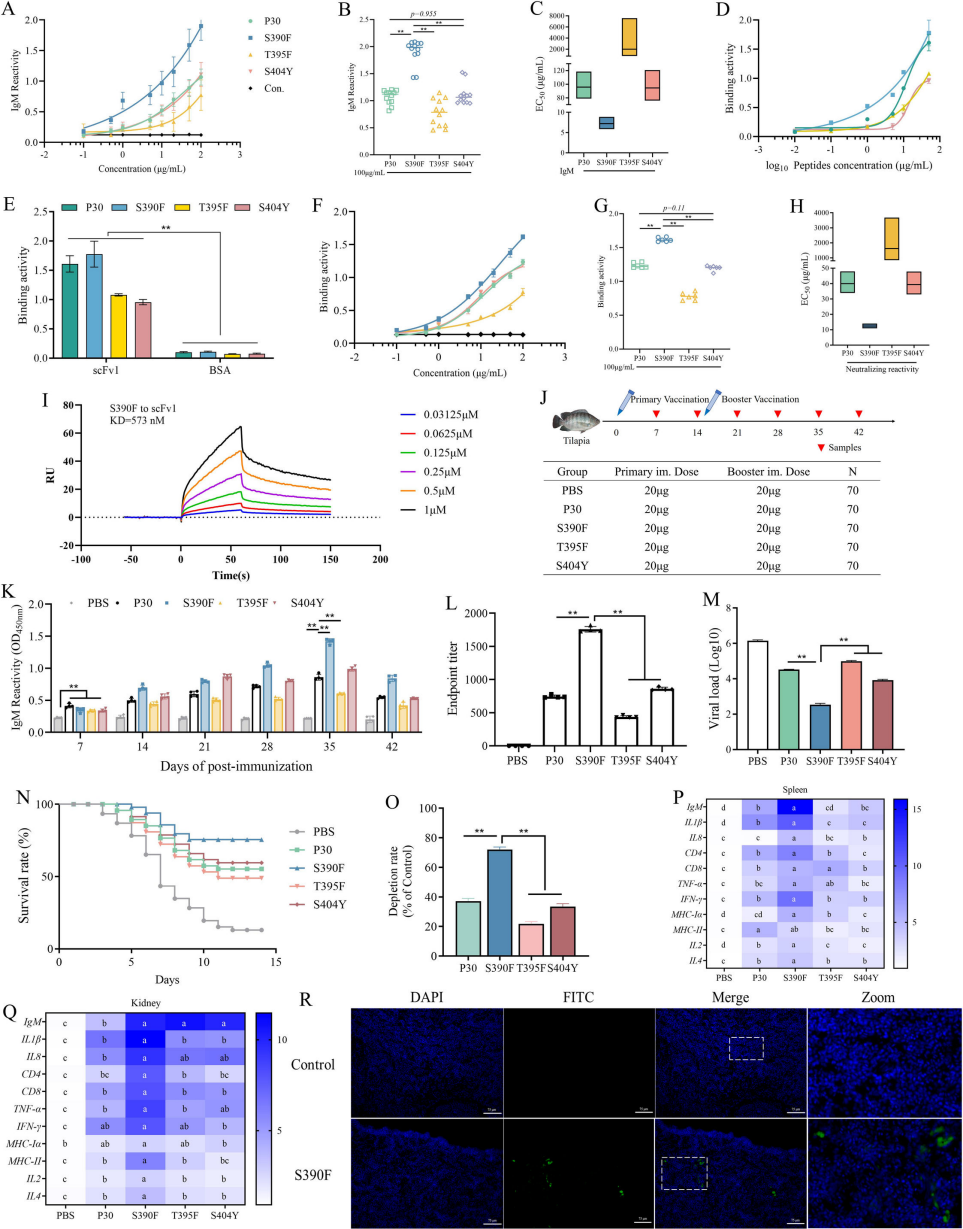
Analysis of affinity and immunogenicity of the designed epitope vaccine. (**A**) The affinity of the epitope peptide to TiLV positive serum was determined by indirect ELISA. The epitope peptide P30 and the mutant peptides S390F, T395F, and S404Y were synthesized by solid phase method and diluted to different concentrations ranging from 0.1 to 100 μg/mL. The data were presented as the mean ± SD. (**B**) Binding ability of the epitope peptide to TiLV-positive IgM at a concentration of 100 µg/mL. **P < 0.01. (**C**) Analysis of the EC_50_ of the epitope peptide in binding to TiLV-positive IgM. (**D**) After obtaining the scFv1 protein using the CHO cell expression system, the binding ability of scFv1 (50 µg/mL) to epitope peptides (P30, S390F, T395F, and S404Y) at different concentrations (0.01–50 μg/mL) was determined by indirect ELISA. Three replicates were performed. (**E**) The BSA protein with the same concentration was used as a negative control, and its binding ability to the epitope peptides was analyzed by indirect ELISA to reduce the possibility of non-specific binding between the antibody and the epitope peptides. Three replicates were set up, and **P < 0.01. (**F**) The binding ability of various concentrations of epitope peptides and scFv1 obtained through prokaryotic expression was determined by indirect ELISA. (**G**) Binding ability of the epitope peptide to scFv1 at a concentration of 100 µg/mL. (**H**) Analysis of the EC_50_ of the epitope peptide in binding to scFv1. (**I**) The binding capacity between the epitope peptide S390F and the purified antibody scFv1 was analyzed using SPR technology. (**J**) The immunogenicity of the epitope vaccine design was analyzed by immunizing tilapia, including two immunization times, sampling times, doses, and sample numbers. (**K**) The serum antibody titers of tilapia were determined at different time points (7, 14, 21, 28, 35, and 42 days) after immunization by direct ELISA. Four biological replicates were set, and the data were presented as the mean ± SD. **P < 0.01. (**L**) The endpoint titer of tilapia serum antibodies 35 days after immunization with the epitope vaccines was determined by direct ELISA. The final titer was the highest dilution multiple at which the serum antibody level was twice that of the negative serum antibody level. Four biological replicates were set, and the data were presented as the mean ± SD. **P < 0.01. (**M**) Spleen tissues were collected from fish that died at 7 days post-TiLV infection to detect the viral load *in vivo*. (**N**) Tilapia were challenged with TiLV 35 days after epitope vaccine inoculation, and the survival rate was calculated. (**O**) Analysis of the exhaustion rate of TiLV-positive antibodies of P30, S390F, T395F, and S404Y epitope peptides by indirect ELISA. The data were presented as the mean ± SD. **P < 0.01. (**P, Q**). The mRNA expression levels of immune-related genes in the spleen and kidney of tilapia at 35 d after immunization were determined by quantitative PCR. The data were presented as the mean ± SD. *P* < 0.05. (**R**) Fluorescence analysis of CD40 in the spleen of tilapia after immunization with the S390F vaccine.

ELISA was used to detect the binding ability of various concentrations of synthetic peptides to TiLV-specific positive serum. The results indicated that the mutant peptide S390F had significantly higher affinity than P30 and other mutant peptides. However, the mutant peptides S404Y and T395F did not show improved affinity compared to P30 (Fig. 7A through C). The results of the affinity between scFv1 obtained from the CHO expression system and the epitope peptides, as determined by indirect ELISA, showed that S390F exhibited stronger binding activity than the wild type and other mutant variants (Fig. 7D), while the BSA control group did not show binding ability (Fig. 7E). This is consistent with the affinity results between scFv1 expressed in *Escherichia coli* by the prokaryotic expression system and the epitope peptides (Fig. 7F through H). Further verification of the binding capacity between epitope peptides and antibody scFv1 was performed using surface plasmon resonance (SPR) technology. The results showed that S390F exhibited a strong binding capacity to antibody scFv1, with a KD value of 573 nM (Fig. 7I).

### S390F epitope peptide induces strong protective immunity.

To evaluate the immunogenicity of epitope peptides designed based on antibody docking, P30 and its mutant peptides (S390F, T395F, and S404Y) underwent primary and booster immunization for a total of 6 weeks. Serum and tissue samples were collected weekly (Fig. 7J). The serum antibody titers of immunized tilapia were determined by direct ELISA at different sampling times. The results showed that peptide immunization promoted a continuous increase in IgM antibody levels in tilapia, reaching a peak at 21 days after booster immunization. The S390F mutant peptide showed significantly better immunogenicity than other peptides, while the T395F and S404Y mutant peptides failed to show significantly improved immunogenicity (Fig. 7K). The IgM endpoint titer induced by the mutant peptides revealed that the S390F immunized group had the highest titer of 1759, which was significantly greater than that of the other groups (Fig. 7L). The results of the TiLV challenge showed that tilapia began to die at 3 days post-infection, accompanied by pathological symptoms such as body darkening and reduced mobility. The mortality rate increased significantly from days 5 to 9 post-infection and persisted until approximately day 11. Epitope vaccine inoculation alleviated this phenomenon. Among the vaccines tested, the S390F epitope vaccine exhibited superior immunoprotective efficacy, with a survival rate as high as 73.3% (Fig. 7N). Seven days post-infection, spleen tissues were collected from dead fish for viral load detection. The results indicated that epitope vaccine inoculation significantly reduced the *in vivo* viral load, and the S390F epitope vaccine showed the optimal protective effect (Fig. 7M).

The TiLV-positive antibody depletion experiments on P30, S390F, T395F, and S404Y demonstrated that the S390F epitope peptide significantly neutralized the TiLV-positive antibodies in tilapia. The depletion rate was approximately 30% higher than that of the P30 epitope peptide (Fig. 7O). Total RNA was extracted from the spleen and kidney of tilapia at the 35-day after immunization to determine the expression levels of immune-related genes. The heatmap results showed that the S390F mutant peptide significantly upregulated all immune genes in the spleen and kidney tissues, among which *IgM* was upregulated by approximately 16-fold. The upregulation of MHC class genes indicates the occurrence of antigen presentation in the organism, while the significant upregulation of *IL2*, *IL4*, *CD4*, and *CD8* confirms the activation of adaptive immune responses and antibody production, suggesting strong vaccine efficacy (Fig. 7P and Q). Furthermore, the study determined the activity of non-specific immune-related enzymes in the serum of tilapia at 7, 21, and 35 days after the initial immunization. The results indicated that the activity of each enzyme in the S390F group was significantly higher than that in the control group and other antigen peptides. The other mutant peptides also significantly increased the enzyme activity level, but there was no significant difference with P30 (Fig. S10). In addition, the immunohistochemical results indicated that after immunization with the S390F epitope peptide, the tilapia kidney tissue was stimulated to express the co-stimulatory molecule CD40, suggesting activation of B cells or dendritic cells (DCs) (Fig. 7R).

**Fig 8:**
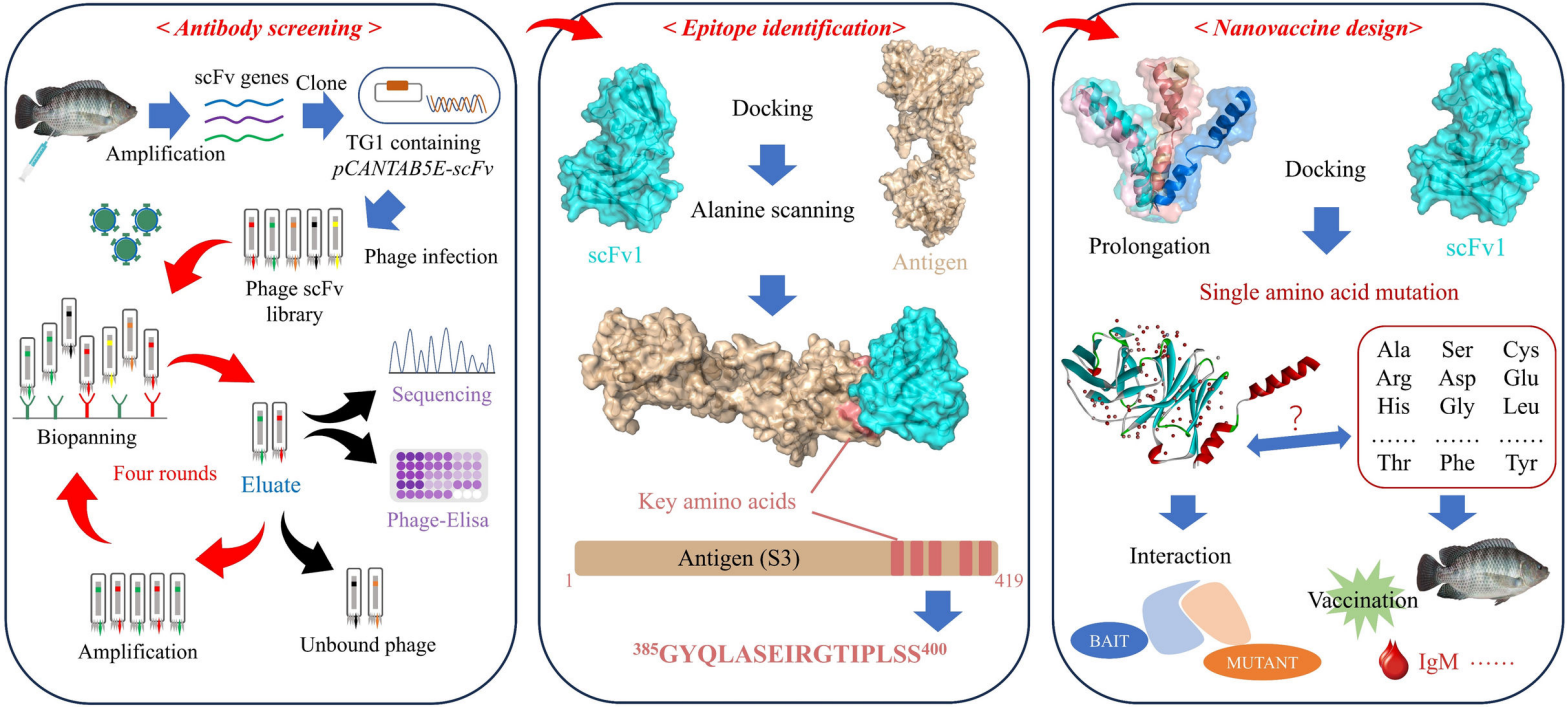
Graphical abstract of this study. Based on the structural characteristics of virus targeting antibodies, we designed an epitope vaccine against tilapia lake virus, which exhibited a potent immune effect.

Overall, this study presents an antibody-based epitope vaccine design *in silico* strategy that elicited a specific immune response with protective effects against TiLV infection. The graphical abstract of the current study is illustrated in Fig. 8.

## DISCUSSION

Viral vaccines are usually designed to direct immune response toward the most conserved epitopes, namely, the neutralizing susceptibility sites of the virus. Compared to subunit vaccines containing larger protein sequences or whole inactivated virus vaccines, epitope-based vaccines have the advantage of triggering a more targeted immune response against key neutralizing epitopes. In recent years, advancements in bioinformatics have significantly propelled the development and broad application of epitope vaccines in the prevention of various diseases (34). Researchers found the fusion peptide–directed antibody, produced by the epitope vaccine designed based on the N-terminus of the HIV-1 fusion peptide, can neutralize various HIV-1 strains. The N-terminus of the HIV-1 fusion peptide is considered a promising target for vaccine development through conformational relationship analysis (35). Vaccines for SARS-CoV-2 are strategically designed to target the dominant epitope regions, aiming to elicit a robust immune response against the virus (36, 37). Crucially, during the design or optimization of an epitope vaccine, enhancing its binding affinity for pathogen-induced specific antibodies is a key strategy for improving vaccine efficacy. Generally, the failure of virus-neutralizing antibodies indicates that the specific viral epitope targeted by the antibody has mutated, rendering the antibody ineffective. Redesigning the virus-neutralizing antibody is often necessary (38). In other words, the epitopes utilized to screen neutralizing antibodies may be crucial to viral immunogenicity and potential as candidate vaccines to prevent viral infection. Therefore, identifying the dominant epitopes and developing corresponding vaccines could offer a swift and potent countermeasure to the initial stages of viral outbreaks.

The 2018 Nobel Prize in Chemistry recognized the achievements of phage display technology in peptides and antibodies, bringing widespread recognition to the phage display platform and highlighting its importance in the field of biotechnology (39). Phage display technology is favored not only for its screening efficiency but also for its inherent advantages in leveraging genetic diversity and facilitating antibody library optimization (40, 41). It finds broad applications in the screening and development of peptide and protein drugs, as well as receptor-targeted protein screening (42, 43), particularly for the screening of animal-toxic, immune-tolerant, and weakly immunogenic antigens (44, 45). Using phage display technology, two BFT nanobodies were obtained from the alpaca immune antibody library, and the potential epitopes Nb2.82 and Nb3.27 of BFT were analyzed by crystal X-ray diffraction (46). A high-sensitivity scFv with a minimum detection limit of 0.03 µg/L was obtained by constructing a MC-LR rabbit immune phage antibody library and screening (47). Rabbit-derived antibodies are commonly used to improve broad-spectrum and reaction sensitivity in antibody preparation. However, the use of heterologous antibodies can present challenges, including potential immunogenicity issues, as well as concerns regarding stability and safety (48, 49). In this study, we constructed an immune library with a capacity of 4.06 × 10^7^ against TiLV primarily using Nile tilapia, highlighting the importance of low heterologous risk, high adaptability, and long antibody half-life in tilapia. Compared to the natural antibody library, the immune library containing a large number of natural TiLV-antibodies produced by tilapia under virus infection is more suitable for constructing and screening of specific antibody candidates, often providing stronger affinity. In parallel, the Fr1 and 4 of tilapia immunoglobulin VH and VL were systematically analyzed and broad-spectrum primers were designed to ensure the diversity of the antibody library from a genetic perspective and provide the possibility of screening high-affinity antibodies (50).

Reverse vaccinology involves identifying dominant viral epitopes by analyzing the binding affinity and stability of antigen-antibody complexes (51, 52). In order to assess the contribution of amino acid residues on the antigen that bind to the antibody during docking, a mutation approach was employed. Specifically, the binding affinity of alanine-substituted mutant antigens for scFv was evaluated. This alanine scanning mutagenesis involved substituting non-essential residues to determine the criticality of specific binding sites. The importance of particular residues in the binding process can be ascertained by comparing the binding affinity and interaction characteristics of the mutant with the antibody to that of the wild-type antigen (53, 54). In a study on the dengue vaccine, researchers identified six epitopes in the dengue envelope proteins targeted by neutralizing antibodies using mutant library and alanine scanning methods (12). In the present study, we applied this approach to demonstrate that the 385–400 site on the S3 antigen contributed most to antibody docking and may represent a potential dominant epitope. The scFv1 is a TiLV-specific antibody obtained from the immune antibody library constructed from natural antibodies after infection. Therefore, an enhanced epitope vaccine, designed based on the scFv1 interaction, is hypothesized to be more effective at conferring protection against viral challenge. This similar strategy for discovering epitopes was also reported in a previous study on rhabdoviruses (13). In the docking model of the S3 antigen and scFv1 antibody, the contribution sites (386, 393, 398, 404, and 409) were distributed on the surface of the structure in both α helix and loop regions, forming a bulge that facilitates the embedding and binding of the antibody.

The conformational dependence of peptide vaccines is a critical design consideration, as it influences the efficiency of antibody recognition and, consequently, the functional efficacy of the epitope in disease protection (34, 55). Therefore, designing peptide vaccines with a more reasonable structure is crucial to induce the activity of the antibody. Studies have shown that epitope peptides identified from the serum of patients with rheumatoid arthritis exhibit a distinct complementary affinity for their autoantibodies (56). To enhance the specific binding of the target epitope, we introduced random single-point amino acid mutations into the P30 peptide. This aimed to spatially alter its binding mode with the scFv1 antibody and improve binding affinity. Among them, mutants S390F, T395F, and S404Y showed great binding activity. Further, the S390F epitope vaccine showed superior affinity with the positive serum produced by natural infection of the virus, indicating that the epitope vaccine may represent the neutralizing susceptible epitope or highly conserved epitope of the virus, which can provide stronger immune protection. A previous study on SARS-CoV-2 targeted the key epitope of the RBD, specifically the 473–488 sites, with the neutralizing antibody CC12.1, as confirmed by molecular dynamics simulations and formed a stable conformation. The critical role of specific binding was confirmed by calculating higher binding energy after point mutation of key epitope to develop advanced antibodies (57). The α helix, β chain/extension, and loop conformation represent the structural forms of the epitope peptide that bind to the antibody (58). Specifically, the amino acids at positions 390, 395, and 404 in the P30 epitope peptide bind to the scFv1 antibody in a helical manner. Unfortunately, *in silico* simulations primarily provide information on changes in binding energy following mutation, reflecting binding strength but not directly visualizing structural differences in the docking complex. Additionally, SPR technology, as the gold standard for verifying antigen-antibody binding, has been widely applied (59). SPR detection revealed that the epitope peptide S390F has a good binding ability to the antibody scFv1 with a dissociation constant of 573 nM, which further validates the feasibility of designing epitope peptides based on antibody conformation.

Since peptide vaccines aim to mimic the surface protein structures of pathogens, the study describes the design of a 30-amino-acid epitope vaccine that incorporates the key epitope region responsible for eliciting an immune response during TiLV infection (60). In a study examining the efficacy of a peptide vaccine in treating HPV, the vaccine demonstrated enhanced internalization and prolonged retention in dendritic cells, which was attributed to its favorable structural features (61). In addition, as a classic immunoadjuvant, Freund’s adjuvant is widely used in vaccine inoculation for animal experiments. It plays a role in promoting the slow release of antigens and activating the immune response (62). In this study, the epitope vaccine, loaded with Freund’s adjuvant, was employed for the immunization of tilapia, with the objective of compensating for the ease with which naked peptides can be removed and of promoting a full immune response in the epitope vaccines.

Immunological results demonstrated that the optimized S390F epitope vaccine exhibited excellent immunogenic activity, inducing strong humoral immune responses and significantly enhancing levels of non-specific immune enzymes. The expression levels of genes encoding certain cellular immune factors were higher than those induced by the unoptimized P30, suggesting a more robust activation of adaptive immunity. The CD40 receptor and its ligand CD40L serve as crucial stimulatory immune checkpoint markers. In this study, the stimulation of CD40 expression by the S390F epitope peptide indicates that dendritic cells are activated for antigen presentation to promote an immune response (63, 64). Additionally, there are still some points to be improved in the present study. First, the broad-spectrum protective effect of the designed vaccine against the virus has not yet been evaluated in the current development of epitope vaccines. However, the genotype of TiLV is relatively conservative and faces few challenges, such as evolution, mutation, and escape. Secondly, this study focuses on presenting a feasible and optimized strategy for designing epitope vaccines based on antibody structure, and on elucidating the activation of immune cells and factors by the S390F epitope peptide. This provides a foundation for subsequent research on the mechanisms of vaccine-induced immune cell responses. The study could benefit from further consideration of the aforementioned challenges.

## MATERIALS AND METHODS

### Fish, virus, cell, and peptides

Healthy Nile tilapias (*Oreochromis niloticus*) in this experiment were supported by fish farms in Guangdong (China), and the breeding and experimental protocols were complied with the guidelines of the Animal Ethical and Welfare Committee, Northwest A&F University. The diagnosis of TiLV was based on PCR testing of the liver, spleen, and kidney of fishes to ensure that the tilapia used in this experiment were TiLV-free. The TiLV (YJ-2021) used in this study was kindly provided by Dr. Zhu from Southwest University and has been preserved in our laboratory (65). HEK293T cells were cultured at 37°C in 5% CO_2_ in Dulbecco’s modified Eagle’s medium (DMEM, Hyclone) with high glucose, supplemented with 10% fetal bovine serum (FBS, TransGen) and penicillin (100 U/mL)/ streptomycin (100 µg/mL). CHO cells were cultured at 37°C in 5% CO_2_ in DME/F12 medium (Hyclone), and CIK cells were cultured in M199 medium (Hyclone) at 28°C, with the rest of the culture conditions remaining the same. Peptides S1WT, S1MuT, S3WT, S3MuT, S10WT, S10MuT, P30, S390F, T395F, and S404Y were synthesized using solid-phase (purity of >85%) by SynthBio Co., Ltd. (Anhui, China) and dissolved in PBS buffer for convenient use.

### Phage display scFv library enrichment and bio-panning

The phage scFv library obtained through M13KO7 infection and PEG/NaCl precipitation was utilized for TiLV-specific bio-panning. TiLV as an antigen was screened and enriched in four rounds in a phage antibody library. Phage titers were measured for each round to calculate the phage fold enrichment. 100 µL virus (2.0 × 10^5^ copies, decreased the volume of antigen by 25 µL for each round) was coated overnight at 4°C in the enzyme-labeled plate. Subsequently, the phage scFv library was mixed with 1% BSA in 3:2 phosphate buffer. After 2 h blocking at 37°C, the phage was bound to the antigen for 2 h and the plates were washed 10 times for 1 min each time with PBST. Glycine-hydrochloric acid (pH 2.2) was then added to gently elute for 10 min and immediately neutralized with Tris-HCl buffer (pH 7.4, Solarbio). The eluted phages were infiltrated with TG1, and the phage yield for this round was calculated in SOB solid medium. The remaining substance was kept at 4°C for amplification and subsequent screening. Four rounds of bio-panning were performed in this experiment, and the multiplicity of enrichment was calculated by determining the input and output phage titers for each round of panning.

### Indirect ELISA was used to analyze the affinity of scFv for TiLV and antigenic peptides

The scFv affinity assay was performed by indirect ELISA. The virus (4 × 10^3^ copies/μL) was incubated overnight at 4°C after being coated in the wells. After blocking with 5% skim milk, 100 µL of scFv1 and scFv3 proteins at the same concentrations (0.01–100 μg/mL) were added and incubated. Meanwhile, BSA proteins with the same concentration gradient were used as controls. The primary and secondary antibodies were 6×His tag mouse mAb and HRP-conjugated goat anti-mouse IgG, respectively, following the previous protocol. The color development and readings were performed as described above. The scFvs obtained from both the prokaryotic and eukaryotic expression systems were verified for their affinity to TiLV. Meanwhile, the BSA protein was used as a negative control.

The indirect ELISA was used to determine the affinity of synthetic peptides for the scFv1 protein and TiLV-positive serum. For the affinity detection of scFv1, the synthetic peptides were diluted at different concentrations (0.01–100 μg/mL) and coated in a microplate. After blocking, scFv1 protein (50 µg/mL) was added and incubated for 2 h at 37°C. In order to reduce the interference of non-specific binding, 50 µg/mL of BSA protein was used as a control for the analysis of binding to the antigenic peptides. Subsequently, 100 µL of 6×His-tag mouse mAb was added to each well and incubated for 1 h, followed by the addition of 100 µL of HRP-conjugated goat anti-mouse IgG for another hour. The color development and OD measurements were performed as described previously. For the affinity detection of serum, 100 µL of positive serum (1:200) was added to each well. The primary antibody was then replaced with mouse anti-tilapia IgM mAb (Frdbio), while all other steps remained unchanged.

### Analysis of antigen-antibody docking interfaces

To assess the impact of amino acid residues on the docking process, the alanine single-point mutation method was utilized. The effect of each site on the docking was determined by measuring the change in binding energy resulting from the mutation. G (wild type) less than G (mutant) represents a positive contribution of this amino acid to antigen-antibody docking. In addition, the amino acid residues and forces of the docking interface were plotted and analyzed in PyMOL. Analysis of the contribution of bound amino acid residues to docking further assists in the exploration of potential epitopes on antigens.

### Immunization

Epitope vaccines usually need to be modified with Freund’s adjuvant (Beyotime) before immunization. Briefly, for a single immunization, the epitope peptide is mixed with an equal dose of complete Freund’s adjuvant and then administered to tilapia. For booster immunization, an equal dose of incomplete Freund’s adjuvant is fused with the epitope peptide before vaccination. For the S1, S3, and S10 epitope vaccine immunization programs, healthy *Oreochromis niloticus* were divided into six groups (70 fish per group) and vaccinated with 20 µg of peptide vaccine by intramuscular injection. During the 1–4 week immunization period, we collected four fish serum samples weekly for immunological analysis.

In the immunization experiments based on the P30-designed vaccine, healthy Nile tilapia were divided into four groups (70 fish per group) and injected intramuscularly with 20 µg of peptide vaccine to complete the primary immunization. After 14 days, the same dose of vaccine was administered again to complete the booster immunization. Serum and tissue samples were collected from 4 fishes in each group weekly after the primary immunization, for a total of 6 weeks. Furthermore, a PBS control was incorporated into both immunization experiments, and the immunization process was conducted in accordance with the aforementioned methodology.

### Analysis of the binding ability between S3 epitope peptide and scFv1 by Western blot

The PET-N-GST-scFv1 plasmid was constructed using seamless cloning technology (Vazyme), and the protein was obtained through a prokaryotic expression system. The scFv1 protein was purified using GST resin (Beyotime) and stored after lyophilization. Similarly, S3-WT, S3-MuT-^Y^386^A^, S3-MuT-^R^393^A^, S3-MuT-^L^398^A^, and S3-MuT were expressed in prokaryotes using the pET32a plasmid. In the Western blot experiment, after blocking, the PVDF membrane containing GST-scFv1 blots was incubated with each of the five S3 proteins at 37°C for 2 h. Subsequently, the anti-6×His tag mouse mAb was used as the primary antibody, and HRP-conjugated goat anti-mouse IgG was used as the secondary antibody. The ECL color development steps were performed as described above.

### Analysis of the depletion of TiLV-positive antibodies by epitope peptides

The epitope peptide to be detected was coated on the ELISA plate at 37°C for 2 h. After washing with PBST, it was blocked with 2% skim milk powder for 2 h to reduce non-specific binding. The TiLV-positive antibody was diluted 800-fold and added to the wells at 100 µL per well. After incubation at room temperature for 20 min, the liquid was aspirated. The ELISA plate was briefly washed, and then, the serum was added again. This process was repeated 10 times, and then, the serum was collected. An ELISA was performed on the collected serum to detect the level of tilapia IgM. Blank and non-depleted negative controls were set up in the experiment. The depletion rate was calculated as follows: Depletion rate = (OD_450_ of non-depleted sample − OD_450_ of detected sample) / OD_450_ of non-depleted sample × 100%.

### Serum antibody levels

Serum antibody levels after immunization were determined by indirect ELISA. Specifically, serum samples were diluted appropriately, coated to enzyme-labeled plates, and incubated overnight at 4°C. After being washed three times with PBST buffer, each well was incubated with mouse anti-tilapia IgM mAb (1:5,000) at 37°C for 1 h. After washing, each well was incubated with HRP-conjugated goat anti-mouse IgG (1:5,500) for an additional hour. Color development was carried out as described above.

### Structure-based *in silico*-designed epitope vaccine

The validation of crucial amino acid sites in the S3 and the results of the simulated peptide immunogenicity indicate multiple hydrophobic or polar amino acids aid in the interaction of S3 to scFv1. We generated a peptide, P30, from S3 and introduced a single amino acid mutation at a specific point to alter its structure for enhancing compatibility with scFv1. In brief, five models of P30 were obtained through predictions using AlphaFold2. Each model was then subjected to docking with scFv1 to identify three different poses with higher ratings, suitable for single-point amino acid mutation. Out of the mutation results, those with a mutation energy lower than −2 were selected for further analysis. The frequency of occurrence of amino acid mutation types at each point was examined to determine the optimal single point mutated peptides for P30.

### Expression of immune-related genes

The expression of immune-related genes (*IgM*, *IL1β*, *IL8*, *CD4*, *CD8*, *TNF-α, IFN-γ*, *MHC-Iα*, *MHC-II*, *IL2*, *IL4*) in tissues at 35 days post-vaccination was determined by qPCR. Total RNA was extracted from the spleen and kidney of immunized tilapia by TRIzol reagent and reverse-transcribed into cDNA as a template for gene detection. Specific primers were used to analyze the expression of immune-related genes by qRT-PCR guided by AceQ qPCR SYBR Green Master Mix (Vazyme) on a CFX96 Real-Time PCR Detection System (Bio-Rad). Gene expression was normalized by the reference gene, *β-actin*. The specific primers for each immune-related gene are listed in Table S3. The amplification procedure of qPCR was: 95°C for 30 s, 60°C for 1 min, 40 cycles. The relative mRNA expression was analyzed by 2^-ΔΔct^ methods.

### Co-Immunoprecipitation

Protein samples were extracted following the co-transfection of HEK293T cells with plasmids containing P30, S390F, G394K, and scFv1 for 48 h, respectively. Subsequent Co-IP assays were performed using the kit (Beyotime) in accordance with the provided protocol. In brief, TBS-washed protein A + G magnetic beads were incubated with GFP-tagged IgG2a mAb (1:3,000, Proteintech) for 1 h at room temperature. Subsequently, the samples were incubated at 4°C overnight. Following the washing of the beads with cell lysates containing protease inhibitors, protein elution was completed with SDS-PAGE loading buffer (Solarbio) for protein blotting. In the Western blot analysis, the GFP-tagged IgG2a mAb (1:3,000) and the Flag-tagged mouse mAb (1:2,000, Beyotime) were employed to identify the IP and input protein samples, respectively. Furthermore, all samples were characterized with a reference protein antibody (β-actin) as a standard control. Enzyme-labeled secondary antibodies consisted of HRP-conjugated goat anti-mouse IgG (1:4,000). The bands were visualized using the ECL Plus hypersensitive luminescent solution (Solarbio) and photographed with a chemiluminescence program. In addition, the His tag system was also used to verify the interaction between epitope peptides, such as P30 and scFv1. Plasmids encoding P30, S390F, and G394K fused with His tags and the plasmid encoding scFv1 fused with a Flag tag were co-transfected into HEK293T cells, respectively. The His-tag monoclonal antibody (1:500, Proteintech) was bound to A + G magnetic beads and used to detect the interaction, and all other operation steps were performed as described above.

In addition, the binding ability of the epitope peptides P30, S390F, and G394K expressed in HEK293T cells to scFv1 was further verified by Western blot *in vitro*, following the procedures described above. Briefly, after transferring the Flag-scFv1 protein onto the PVDF membrane, His-P30, S390F, and G394K peptides were incubated with the PVDF membrane at 37℃ for 2 h. Subsequently, the samples were labeled with a His-tag monoclonal antibody followed by an HRP-goat anti-mouse antibody and developed using ECL. At the same time, β-actin was used as an internal reference, and a BSA control group was set up.

### Yeast two-hybrid assay

The pGBKT-scFv1 bait plasmid was transformed into Y_2_Hgold yeast competent cells (WEIDI) and cultured in SD-deficient medium (Beijing Zoman Biotechnology Co., Ltd.) to confirm the absence of self-activation. Subsequently, pGADT-P30, pGADT-S390F, and pGADT-G394K plasmids, along with the pGBKT-scFv1 bait plasmid, were co-transformed into Y_2_Hgold yeast competent cells. The cells were then cultured on LT-deficient SD and LTHA-deficient SD medium for 48–96 h to observe the expression of the reporter genes. Additionally, the pGADT-T plasmid was co-transformed with the pGBKT-53 and pGBKT-lam plasmids and cultured as positive and negative controls, respectively.

### Cellular co-localization

The pEGFP-P30, S390F, and G394K plasmids, along with the pCMV-C-DsRed-scFv1 plasmid, were co-transfected into HEK293T cells and cultured for 48 h. Afterward, the cells were fixed with 4% paraformaldehyde, permeabilized, and stained with DPAI (Beyotime), which contains an anti-fluorescence quencher. The expression and co-localization of proteins in cells were observed using confocal fluorescence microscopy.

### Immunohistochemical analysis of CD40 expression in tilapia

The spleen tissues of tilapia were collected 35 days after immunization, fixed with 4% paraformaldehyde, and sent to Y&K Bio company (Shaanxi, China) for the preparation of frozen sections, which were stored at −20°C. The tissue positions in the sections were marked with a histochemical pen, and the section positions were covered with immunol staining blocking buffer (Beyotime) and blocked at room temperature in a wet box for 2 h. Subsequently, CD40 rabbit monoclonal antibody (1:150, Beyotime) was applied as the primary antibody and incubated overnight at 4°C in a wet box. FITC-labeled goat anti-rabbit IgG (1:200, Beyotime) was used as the secondary antibody and incubated at room temperature in a wet box for 2 h. After dropping the immunostaining permeabilization buffer with Triton X-100 (Beyotime) and incubating at room temperature for 10 min, the cell nuclei were labeled with DAPI, and the expression of CD40 was observed using a fluorescence microscope. The sections were washed with PBS between each step. The PBS-immunized group was used as the control in the experiment.

### SPR assay

Protein-ligand interactions were analyzed using a Biacore T200 instrument (Cytiva) with CM5 sensor chips at 25°C. S390F was immobilized on flow cell 2 (FC2) via amine coupling: the chip surface was activated with EDC/NHS (10 µL/min, 7 min). S390F (50 µg/mL in sodium acetate buffer, pH 4.0–5.0) was injected (10 µL/min) until 10,000 RU immobilization was achieved. Residual reactive groups were blocked with ethanolamine (10 µL/min, 7 min). FC1 served as a reference surface. All binding experiments used a running buffer (1× PBS, pH 7.4, 5% [vol/vol] DMSO). Solvent correction curves were generated using DMSO concentrations ranging from 4.5% to 5.8%. Serial dilutions of antibody scFv1 in running buffer were injected over FC1 and FC2 at 30 µL/min for 60 s (association), followed by buffer flow (dissociation). The surface was regenerated with 10 mM glycine-HCl (pH 2.0; 5 min contact time) between cycles. Sensorgrams were reference-subtracted (FC2-FC1) and globally fitted to a 1:1 Langmuir binding model using Biacore Insight Evaluation Software (Cytiva) to derive kinetic constants (Ka, Kd) and affinity (KD).

### TiLV challenge

Three weeks after the booster immunization with the epitope vaccine, the surviving tilapia were challenged with TiLV. Forty-five fish were selected from each group and intraperitoneally injected with 6 × 10^5^ copies of TiLV solution. The water temperature was controlled and maintained at 29 ± 1℃, and mortality was continuously recorded. During the mortality peak period, spleen tissues were collected from diseased fish in each group, and viral copy numbers were detected to evaluate the immunoprotective efficacy.

### Statistical analysis

Statistical analyses of all data were performed using GraphPad Prism version 9.0 (GraphPad Prism Software, USA). Results were presented as mean ± standard deviation (SD). Statistical comparisons were performed using a one-way analysis of variance (ANOVA) with Dunnett’s multiple-comparison test. The significance level was set at *P ≤ 0.05 and **P ≤ 0.01 to indicate statistical significance.

## Data Availability

All data are available within the article and its supplemental material. Source data are provided with this paper.

## SUPPLEMENTAL MATERIAL

Supplemental figures jvi.00689-25-s0001.docx Fig. S1 to S10.

Supplemental methods jvi.00689-25-s0002.docx Additional experimental details.

Tables S1 to S4 jvi.00689-25-s0003.docx Docking residues of scFv1 and antigens, frequencies of mutation types, and primer sequences.

Table S5 jvi.00689-25-s0004.xlsx Complete mutation data of 15 models, with a total of 11,250 mutation results.

ASM does not own the copyrights to Supplemental Material that may be linked to, or accessed through, an article. The authors have granted ASM a non-exclusive, world-wide license to publish the Supplemental Material files. Please contact the corresponding author directly for reuse.
