# Supplementary material for: Design of antibody structure-guided epitope vaccines in silico to induce potent immune responses against emerging viruses
Source: J Virol. 2025 Nov 11;99(12):e00689-25. doi: 10.1128/jvi.00689-25 (PMC12724136; doi:10.1128/jvi.00689-25)
Supplement: Supplemental figures — Fig. S1 to S10. [file jvi.00689-25-s0001.docx]

**Supplemental Material- Figures**


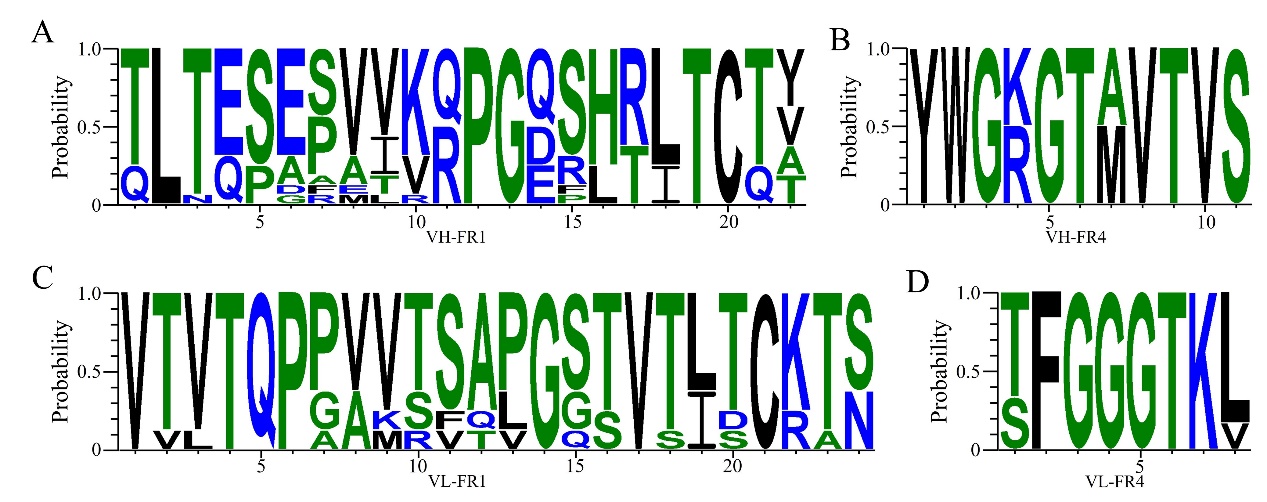


**Figure S1. Amplification and expression of scFv.** **A.** The conservation of the sequences of the VH-Fr1 of tilapia immunoglobulin was analyzed by the Weblogo3 server to design specific primers. **B.** VH-Fr4. **C.** VL-Fr1. **D.** VL-Fr4.


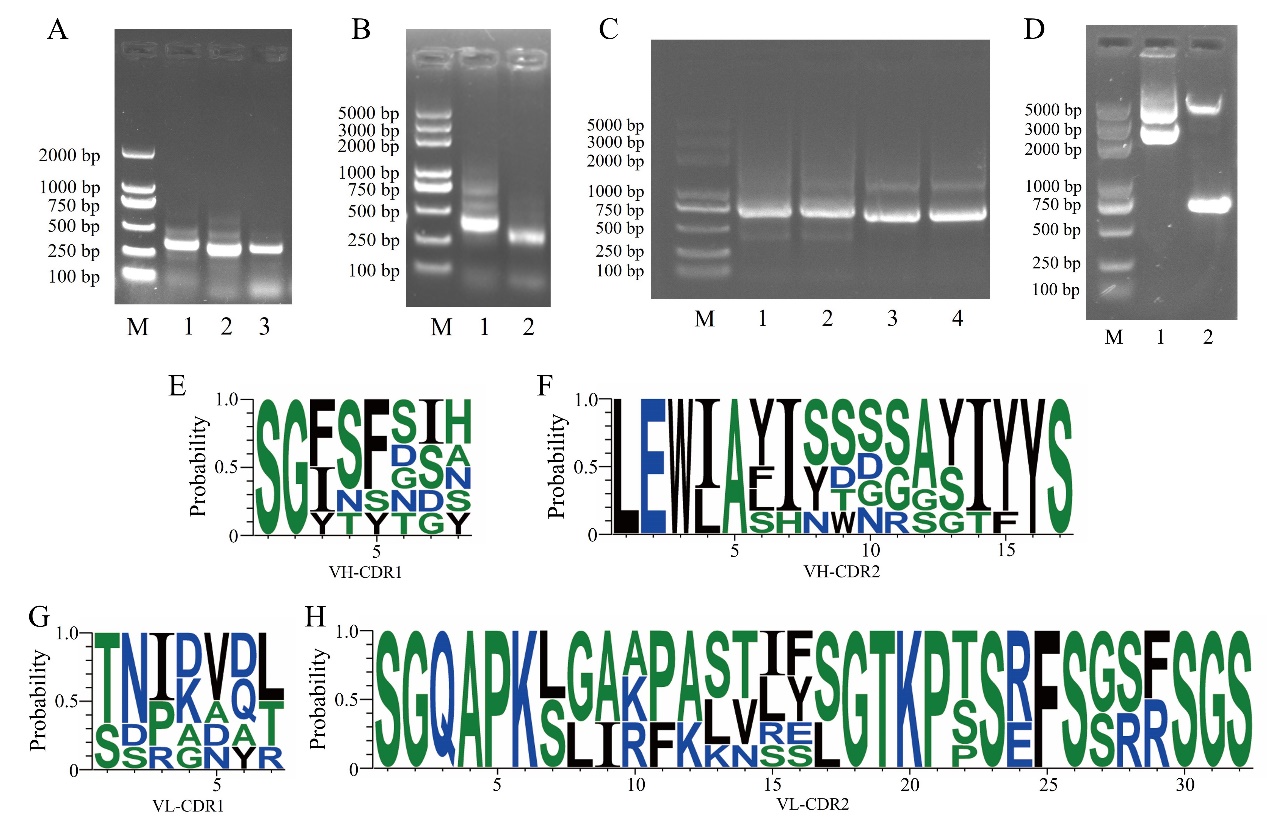


**Figure S2. Amplification and sequence analysis of scFv. A.** The VH genes of tilapia immunoglobulin were amplified by specific primers. The primers are listed in Table S2. Lane M, Marker; Lane 1-3, VH genes. **B.** VL genes. Lane M, Marker; Lane 1-2, VL genes. **C.** The scFv was obtained by SOE-PCR splicing of VH and VL genes using flexible peptides (GGGS×3). M, Marker; 1-4, scFv genes. **D.** The phage plasmid pCANTAB5E-scFv was double digested by *Sfi* I and *Not* I enzymes. Lane M, Marker; Lane 1, complete plasmid; Lane 2, double enzyme digestion plasmid. **E.** Abundance of CDR1 region of scFv VH in antibody library. The monoclonal CDRs sequence in the antibody library was analyzed for richness using the Weblogo3 server after sequencing. **F.** VH-CDR2. **G.** VL-CDR1. **H.** VL-CDR2.


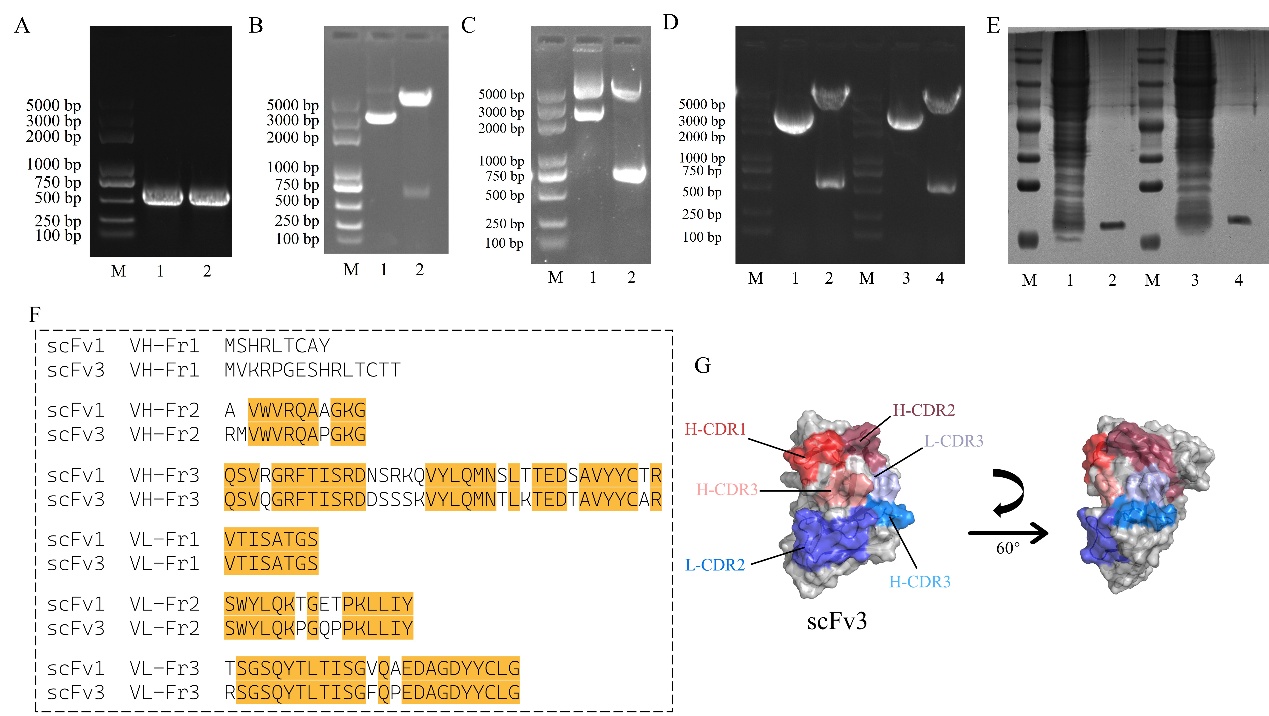


**Figure S3. Expression and amino acid sequence analysis of scFv.** **A.** Amplification of scFv genes containing *BamH* I and *Hind* III restriction sites. Lane M, Marker; Lane 1-2, scFv1 gene; 3, scFv3 gene. **B.** The expression plasmid pET32a-scFv1 was double digested by *BamH* I and *Hind* III enzymes. Lane M, Marker; Lane 1, complete plasmid; Lane 2, double enzyme digestion plasmid. **C.** pET32a-scFv3 plasmid. **D.** The construction of the recombinant plasmids pcDNA3.1-scFv1 and pcDNA3.1-scFv3 was identified by double enzyme digestion (*BamH* I and *EcoR* V). M, Marker; 1, pcDNA3.1-scFv1; 2, scFv1; 3, pcDNA3.1-scFv3; 4, scFv3. **E.** The scFv proteins expressed in CHO cells and purified using the His tag were identified by SDS-PAGE. M, Marker; 1, scFv1; 2, purified scFv1; 3, scFv3; 4, purified scFv3. **F.** Comparison of the amino acid sequences in the framework regions of the scFv1 and scFv3. **G.** PyMOL was used to display the three - dimensional structure of the scFv3 and label the CDRs.


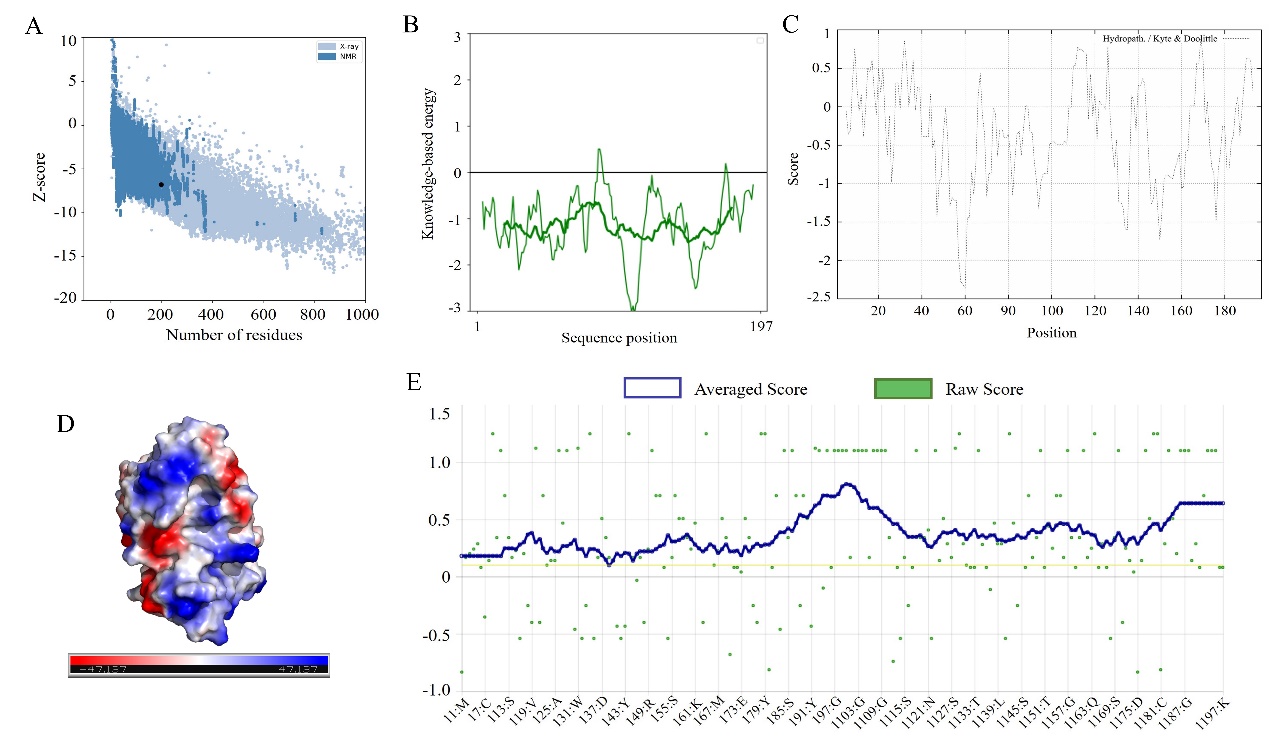


**Figure S4. Sequence and model quality analysis of scFv1.** **A.** The scFv1 models were scored using the SAVESv6.0 tool. **B.** The scFv1 model energy and the amino acid sequence position were plotted by the ProSA tool to analyze the local model quality. **C.** ProtScale tool was used to analyze the hydrophilicity and hydrophobicity of scFv1 sequences. **D.** The surface potential of scFv1 models was analyzed by PyMOL. Red represents low potential and blue represents high potential. **E.** Verify 3D of scFv models analyzed the correlation between the atomic model (3D) and the corresponding amino acid sequence (1D).


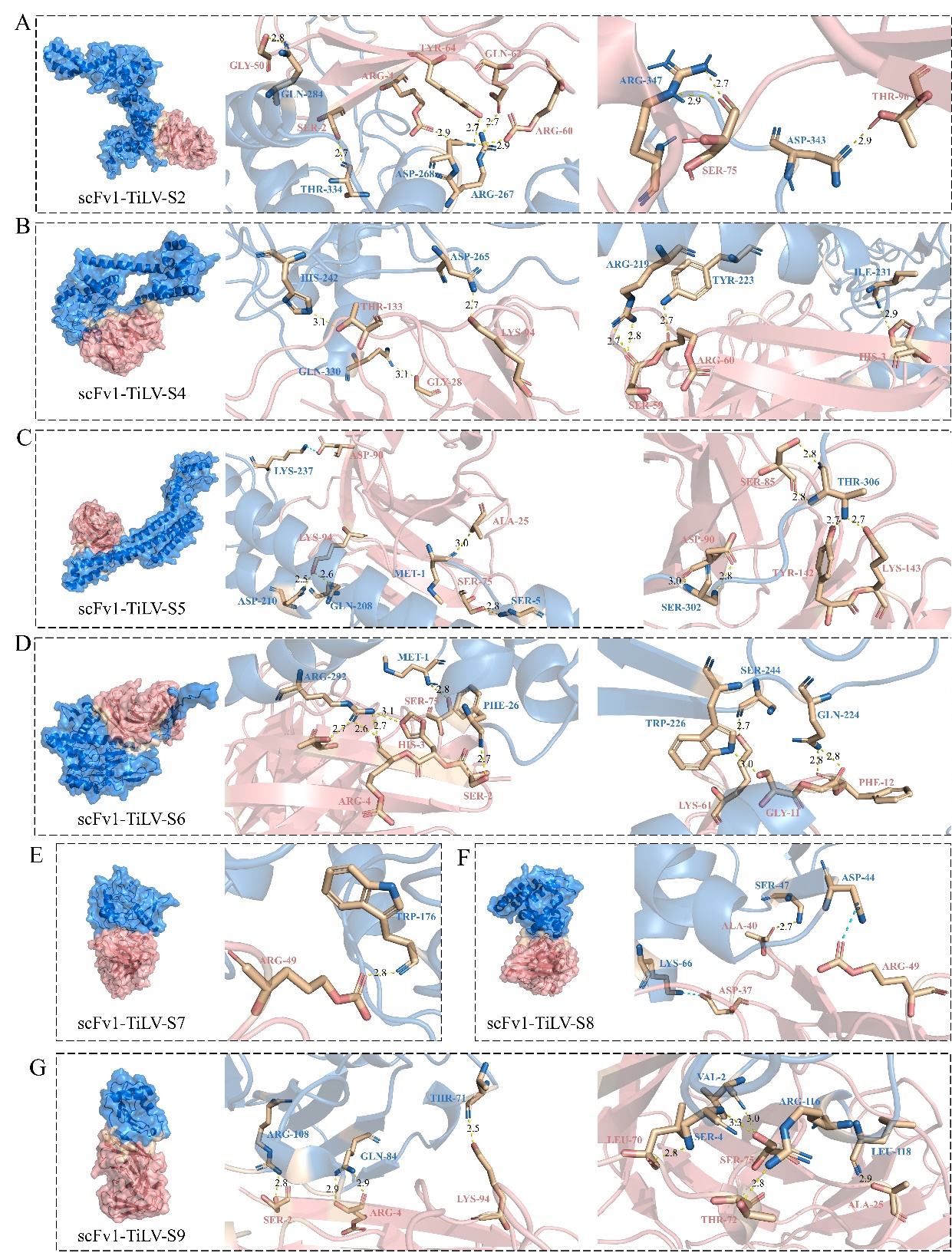


**Figure S5. Three-dimensional docking analysis of scFv1 and antigens.** **A.** Docking model for scFv1 and S2 antigen, with antigens marked Marine, scFv1 marked Salmon, and binding amino acids marked Wheat. The hydrogen bonds and key amino acids in the three-dimensional docking interface between scFv1 and S2 was mapped by PyMOL. **B.** S4 antigen. **C.** S5 antigen. **D.** S6 antigen. **E.** S7 antigen. **F.** S8 antigen. **G.** S9 antigen.


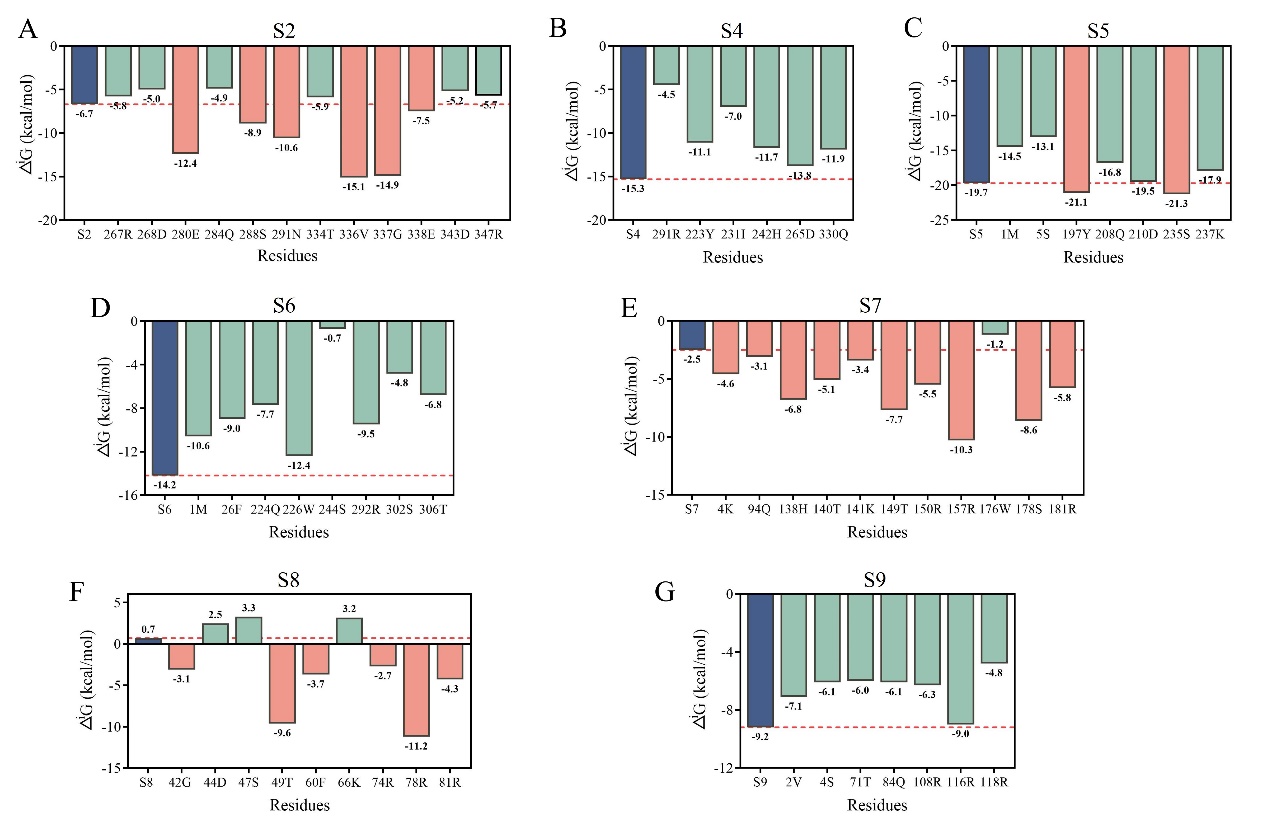


**Figure S6. Analysis of the antigen - antibody binding energy after alanine mutation.** **A.** The alanine mutations were introduced to the amino acids that were binding to the antigen S2, and the binding energy to scFv1 was then recalculated. **B.** S4 antigen. **C.** S5 antigen. **D.** S6 antigen. **E.** S7 antigen. **F.** S8 antigen. **G.** S9 antigen.


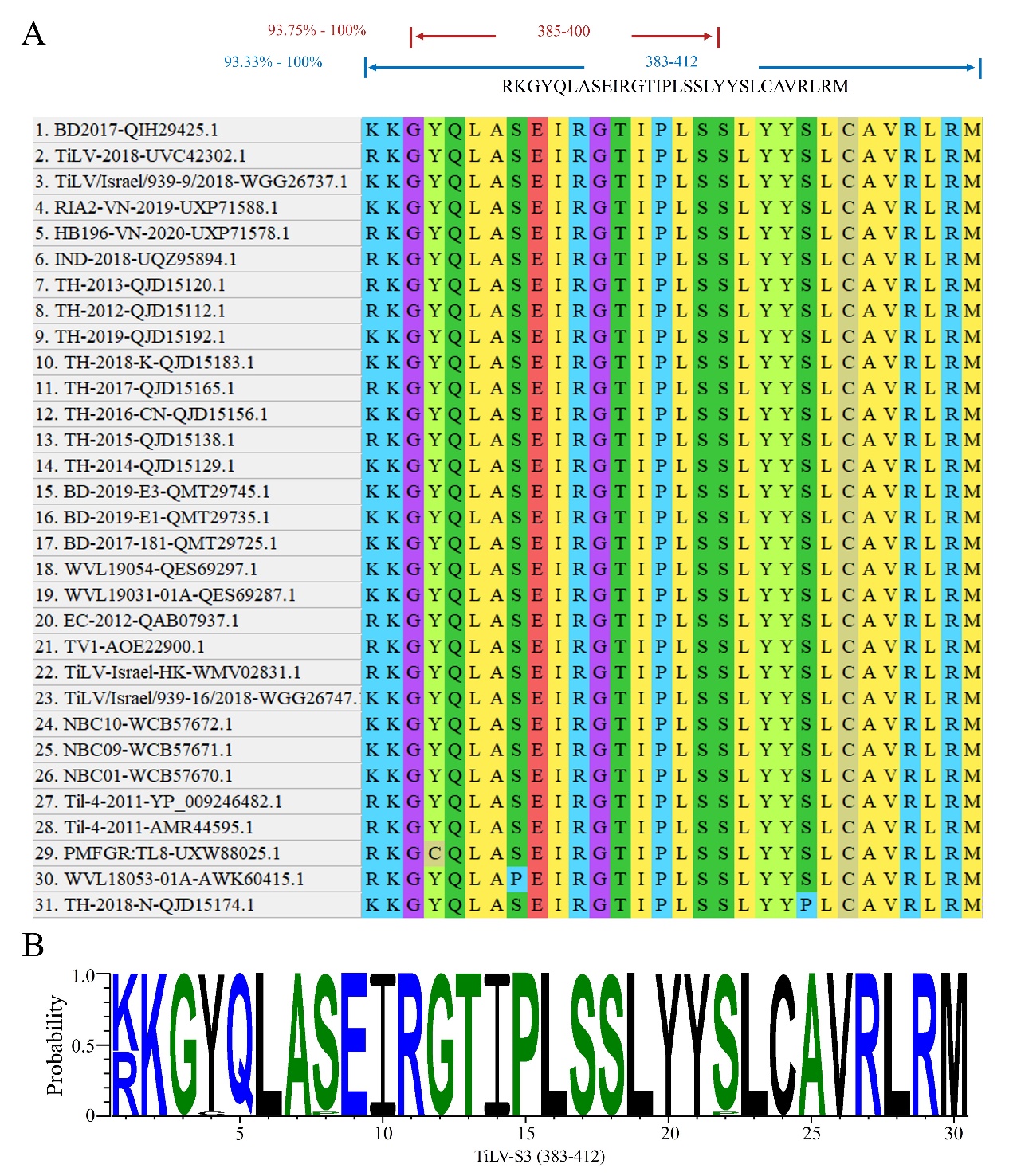


**Figure S7. Conservative analysis of S3 dominant and P30 epitopes.** **A.** Homology of 383-412 sites of antigen S3 in 28 different TiLV strains was analyzed by Mega6. The sequences were provided by NCBI. **B.** The amino acid types of each site were identified by Weblogo3 tool to analyze conservation.


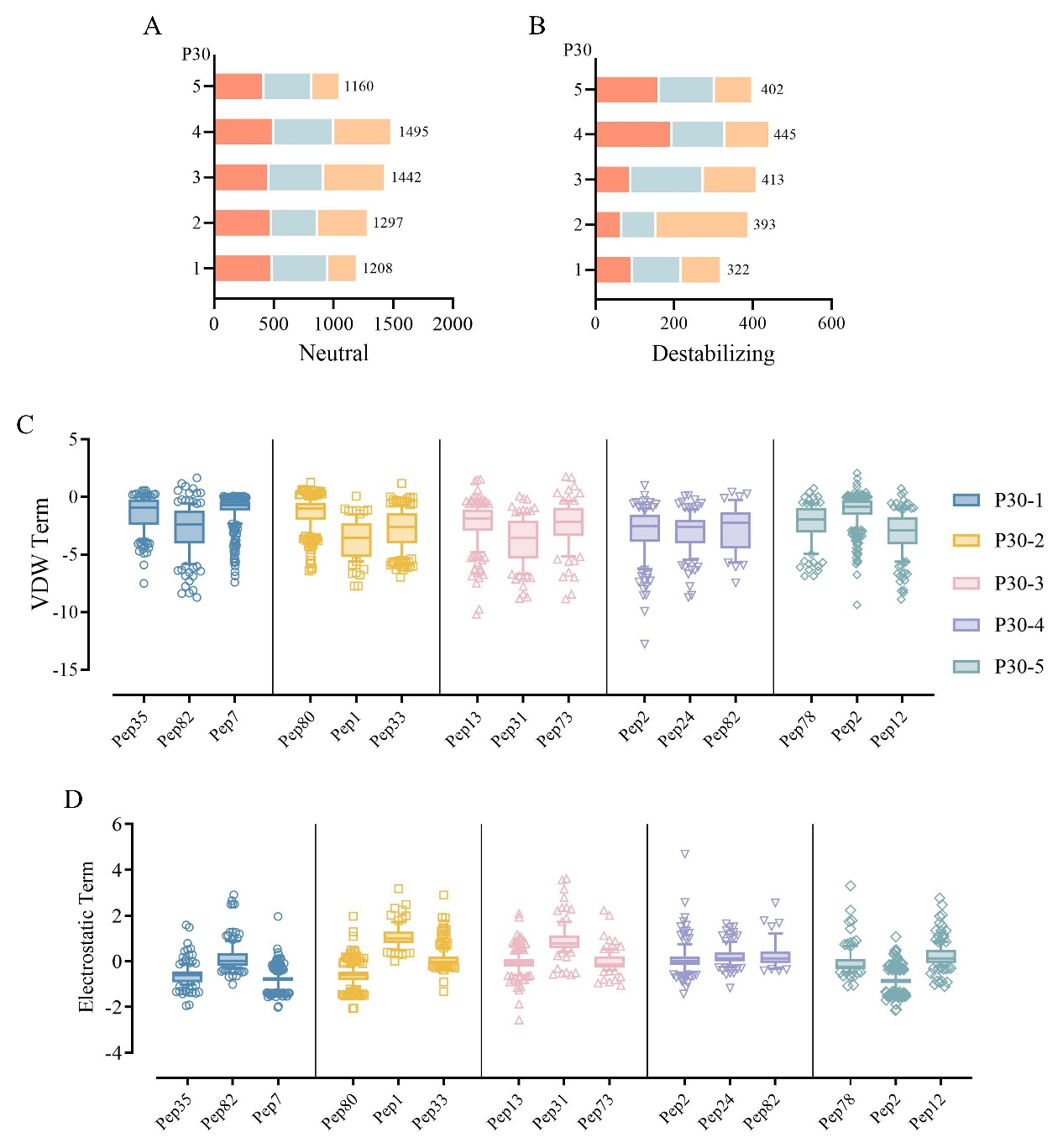


**Figure S8. Single amino acid random mutation analysis of the P30 epitope peptide. A.** The number of neutral mutations generated after single amino acid random mutations in different poses of the five models of the P30 peptide. **B.** The number of unstable mutations generated after single amino acid random mutations in different poses of the five models of the P30 peptide. **C.** The VDW changes after single amino acid random mutations in 15 docking poses. **D.** The electrostatic potential changes after single amino acid random mutations in 15 docking poses.


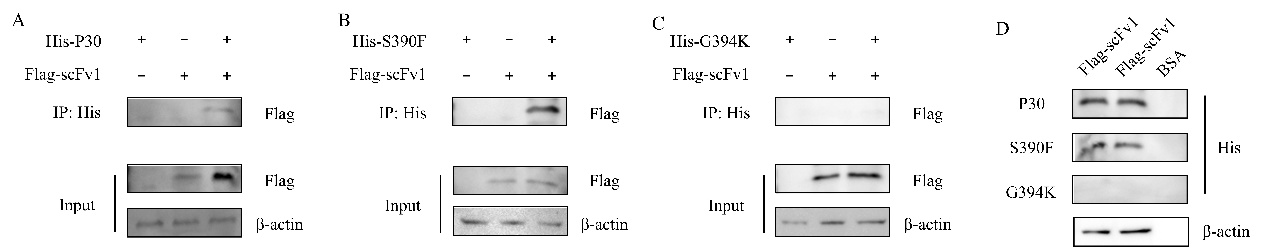


**Figure S9. The interaction between the epitope peptides and scFv1 in the His tag system was identified using CoIP and Western blot methods. A.** P30 peptide and scFv1. The plasmids pcDNA3.1-P30 and pCMV-Flag-scFv1 were constructed and co-transfected into HEK293T cells. After 48 h, the proteins were collected and incubated with magnetic beads treated with a mouse anti-His monoclonal antibody. The samples were eluted with SDS-PAGE loading buffer and verified by Western blot. β-actin was used as an internal reference. **B.** S390F peptide and scFv1. **C.** G394K peptide and scFv1. **D.** The binding ability of scFv1 to the peptides P30, S390F, and G394K was identified by Western blot. The scFv1 was fused with a Flag tag with two replicates set up. The peptides were fused with a His tag. The BSA protein was used as a control, and the β-actin protein was used as an internal reference.


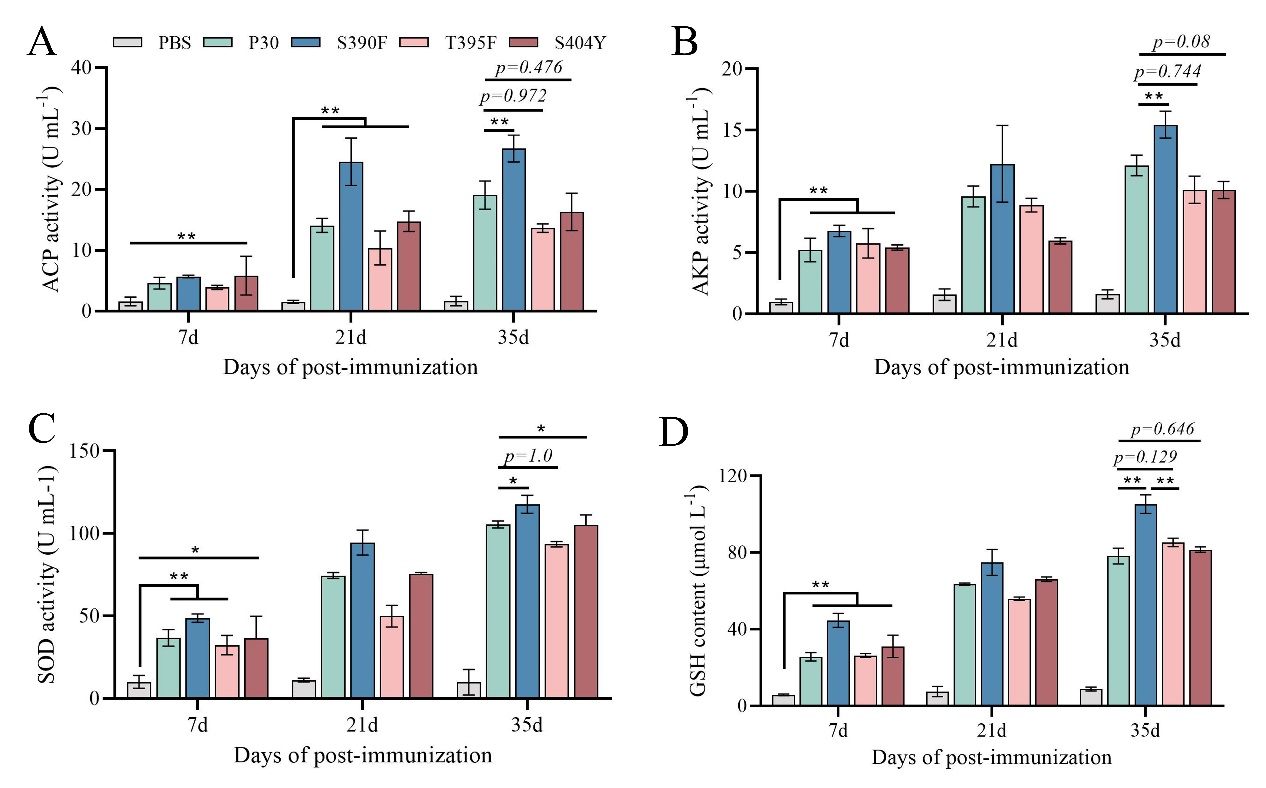


**Figure S10. Analysis of immunogenicity of epitope vaccine.** **A.** The levels of ACP enzyme activities in the serum of tilapia at different times (7, 21, and 35 d) after immunization with the epitope vaccines were determined according to the protocol of the kits. **B.** AKP. **C.** SOD. **D.** GSH. Four biological replicates were set and the data were presented as the mean ± SD. **p*<0.05, ***p*<0.01.
