# Supplementary material for: Design of antibody structure-guided epitope vaccines in silico to induce potent immune responses against emerging viruses
Source: J Virol. 2025 Nov 11;99(12):e00689-25. doi: 10.1128/jvi.00689-25 (PMC12724136; doi:10.1128/jvi.00689-25)
Supplement: Supplemental methods — Additional experimental details. [file jvi.00689-25-s0002.docx]

**Supplemental Material - METHODS**

***TiLV-specific spleen acquisition***

Before constructing the phage library, viral infection was necessary to obtain spleens that tested positive for tilapia. In brief, sera and spleens from TiLV-infected tilapia (5.2 ± 0.3 g) were collected at different times to assess serum antibody responses and to construct a phage antibody library. The serum antibody responses of tilapia were assessed by directly ELISA. The collected serum was diluted with the coating solution to various concentrations and then coated onto the microplate as target molecule. The primary antibody is mouse anti-tilapia IgM mAb (Frdbio), and the secondary antibody is HRP-conjugated goat anti-mouse IgG (Sangon Biotech). Color development was achieved through the use of TMB working solution (Solarbio), and absorbance was subsequently measured at 450 nm using a microplate reader. Furthermore, the serum exhibiting the highest IgM antibody levels after infection underwent additional testing for TiLV-specific antibody levels using indirect ELISA. The virus was coated with 4.0×10^5^ copies, and the subsequent steps were identical to those described above. Meanwhile, total RNA of spleen collected at this time point was extracted by TRIzol method according to the manufacturer’s protocol (Invitrogen) and cDNA was prepared using HiScript II 1st strand cDNA Synthesis Kit (Vazyme).

***Phage display scFv library construction***

Primers were design based on Nile tilapia (*Oreochromis niloticus*) Ig VH and VL obtained from NCBI and synthesized by Sangon Biotech (Shanghai, China). The conservation of the Ig frame regions 1 and 4 were analyzed using the WebLogo 3 server (*https://weblogo.threeplusone.com*). All primers listed in Table S4. The VH and VL genes were specifically amplified by cDNA and ligated into a complete scFv by splicing overlap extension PCR. The full scFv genes (VH-Linker-VL) were ligated by a flexible linker (GGGGS×3), using SOE-PCR with the VH forward and VL reverse primers. The PCR products were verified using 1.5% agarose gel and the correct band-size, approx.750 bp, was purified by kit. The construction of phage display scFv library was carried out as described in a previous study (13). In brief, the scFv gene and pCANTAB5E vector (HonorGene) were double-digested using restriction endonucleases *Not* I and *Sfi* I (New England Biolabs) and ligated by T4 DNA ligase (TaKaRa). Subsequently, the plasmid containing the scFv gene was transformed into *E. coli* TG1 cells (Miaoling Biology) by electro-transformation and infected with helper phage M13KO7 (New England Biolabs) to produce daughter phages. Subsequently, recombinant phages expressing the fusion protein were precipitated with a PEG/NaCl solution to obtain recombinant phages. The transformed TG1 products were cultured in agar 2 × YT medium containing ampicillin and monoclonal phages were selected for amplification to determine the recombination rate of the antibody library. Monoclonal phages were sequenced and analyzed for CDRs. The volume of the phage scFv library was calculated using plate counting and then stored at -80°C until required for use.

***Phage ELISA***

Before performing phage Elisa to detect affinity, the fourth elution product was infected with *E. coli* TG1 and cultured overnight in solid 2 × YT medium. Total 30 monoclonals were picked and expanded for culture. Phage in bacterial solution was infected by helper phage and amplified in 2 × YT medium. ELISA plates were coated with 100 μL TiLV (diluted to 4×10^3^ copies/μL in 0.1 M NaHCO_3_) and incubated at 4°C overnight. After washing three times, each well was incubated with a containment solution (5% BSA) for 2 h at 37°C. The phage clones were added to the antigen-coated wells and incubated for 2 h at room temperature. After washing, 100 μL M13 Major Coat Protein Antibody HRP (diluted 1:4000 in 5% BSA; Santa Cruz) was applied as an enzyme marker antibody to the plate. TMB kit was performed the color development and absorbance was read in 450 nm with the microplate reader. The positive rate of recombinant phage was calculated based on the criterion of exceeding 2.1 times the OD value of the negative control as a positive result (33). Plasmids were extracted for DNA sequencing following expansion of positive phage clones.

***Identification and affinity ELISA analysis of scFv***

DNA amplification of scFv was performed using scFv-F/R primers, respectively. The purified scFv PCR products and pET32a plasmid were digested by *BamH* I and *Hind* III restriction enzyme (TaKaRa). Subsequently, T4 ligase (TaKaRa) was used to ligate the two components at 16°C overnight, and the plasmid containing scFv was transformed into *E. coli* Bl21 for expression at 37°C. Isopropyl-β-D-thiogalacto-pyranoside (IPTG; Sigma-Aldrich Trading Co., Ltd.) was added to a final concentration of 1 mM to induce the protein expression. Subsequently, the bacterial pellets were collected at 8000 rpm. After being dissolved in PBS buffer, the mixture was disrupted for 20 minutes using an ultrasonic probe at a power of 300 W. Then, it was centrifuged at 8000 rpm to denature the protein with 8 M urea. After the protein was completely dissolved, it was sterilized by passing through a 0.22 μm filter membrane and purified using a nickel column (Sangon Biotech). The purified protein was renatured by gradient dialysis and concentrated by lyophilization.

In addition, CHO cells were used for the in vitro eukaryotic expression of the scFv protein. Eukaryotic expression strains of pcDNA3.1-scFv1 and scFv3 were constructed according to the above method, and a His tag was fused to the C-terminus. The CHO cells were transiently transfected with lipo8000 liposomes (Vazyme) and continuously cultured for 24-48 h. Subsequently, the cell proteins were collected using a cell lysate containing pmsf (Beyotime), and the supernatant was collected after centrifugation at 8000 rpm for 5 min. The supernatant was purified using a His-tagged protein purification kit (IDA-Ni agarose magnetic beads, Beyotime) according to the requirements of the reagent manufacturer.

The purified products were analyzed by 12% polyacrylamide gel electrophoresis and Western Blot. The anti-6 × His tag mouse mAb (1:2000, Sangon Biotech) and HRP-conjugated goat anti-mouse IgG (1:4000) were provided as enzyme-labeled antibodies. The remaining steps were identical to those previously described for Co-IP.

***The protection studies of scFv1 and TiLV in vivo***

Healthy Nile tilapia (*Oreochromis niloticus*) with a body weight of approximately 3.2±0.6 g were selected. After being temporarily cultured in a circulating water aquaculture system, they were randomly divided into a neutralization experimental group, a virus control group, and a BSA control group, with 30 fish in each group. In the experimental group, 50 μL of TiLV virus suspension (8×10^3^ copies/μL) was injected intraperitoneally. Twelve hours later, a solution of scFv1 diluted in PBS at a dose of 10 μg/g was injected intraperitoneally. In the BSA control group, after injecting the same dose of TiLV, BSA solution of the same concentration was used instead of the scFv1. In the virus control group, only virus was injected. Meanwhile, a blank control was set up. After the injection, the mortality of tilapia was observed and recorded continuously for 10 days, and the cumulative mortality rate was calculated.

***Analysis of the binding between scFv1 and TiLV by cellular fluorescence***

After being cultured for 48 h, CIK cells grew into a monolayer and were then continuously incubated with TiLV virus at 28°C for 72 h. Subsequently, the culture medium was replaced, and the cells were incubated with GST-scFv1 protein for 24 h to ensure binding to TiLV particles. The cells were fixed with 4% paraformaldehyde at room temperature for 10 min and then incubated with immunostaining permeabilization buffer with Triton X-100 for 10 min. Subsequently, the GST mouse mAb (1:500, Beyotime) was used as the primary antibody and incubated with CIK cells at 4°C overnight, and FITC-labeled goat anti-mouse IgG (1:500, Beyotime) was used as the secondary antibody. After DAPI staining, the cells were observed under a fluorescence microscope. Cells that were not incubated with GST-scFv1 protein served as the control.

***Three-dimensional structure prediction and antigen-antibody docking***

The protein structure of scFv was predicted and modelled online by the Alaphfold2 with default options. The scFv structure were visualized using PyMOL, allowing for the identification and localization of the VH and VL CDRs, which play a vital role in the binding of antigens and antibodies. The method described above was also performed for the structural models of the TiLV S1-S10 for antigen-antibody docking. The Cluspro server (*https://cluspro.bu.edu*) was employed for proteins docking, while the PDBePISA tool (*https://www.ebi.ac.uk/msd-srv/prot_int/*) was utilized to analyze the binding energy and other characteristics of the docking complex. The docking amino acid sites of the complex were shown using the LigPlot.

***Analysis of the Reliability of the scFv1 Model***

The SAVESv6.0 tool (*https://saves.mbi.ucla.edu*) was used to score the scFv1 model. The ProSA tool (*https://prosa.services.came.sbg.ac.at*) was employed to plot the energy and amino acid sequence positions of the scFv1 model for analyzing the local model quality. The ProtScale tool (*https://web.expasy.org/protscale*) was utilized to analyze the hydrophilicity and hydrophobicity of the scFv1 sequence. The surface potential of the scFv1 model was analyzed using PyMOL, where red represents low potential and blue represents high potential. The 3D structure of the scFv1 model was identified to analyze the correlation between the atomic model (3D) and the corresponding amino acid sequence (1D).

***Activities of non-specific enzymes***

At 35 days after primary immunization, acid phosphatase (ACP), alkaline phosphatase acidity (AKP), superoxide dismutase (SOD) and reduced glutathione (GSH) in serum of tilapia in each group were measured using kits (Nanjing Jiancheng Bioengineering Institute) in accordance with the protocol provided by the manufacturer.
