# Supplementary material for: Design of antibody structure-guided epitope vaccines in silico to induce potent immune responses against emerging viruses
Source: J Virol. 2025 Nov 11;99(12):e00689-25. doi: 10.1128/jvi.00689-25 (PMC12724136; doi:10.1128/jvi.00689-25)
Supplement: Tables S1 to S4 — Docking residues of scFv1 and antigens, frequencies of mutation types, and primer sequences. [file jvi.00689-25-s0003.docx]

**Supplemental Material – Tables**

Table S1. The docking residues of scFv1 and antigens

| Antigen | Residues | △^i^G (kcal/mol) | Interface  area, A^2^ |
| --- | --- | --- | --- |
| Segment 1 | 36Glu, 59Glu, 63Arg, 91Thr, 92Leu, 94Arg, 124Lys, 224Asp, 231Asp, 237Ser, 240Val, 247Arg | -9.6 | 1389.0 |
| Segment 2 | 267Arg, 268Asp, 280Gly, 284Gln, 288Ser, 291Asn, 334Thr, 336Val, 337Gly, 338Glu, 343Asp, 347Arg | -6.7 | 1109.9 |
| Segment 3 | 386Tyr, 393Arg, 398Leu, 404Ser, 409Arg | -13.3 | 798.2 |
| Segment 4 | 219Arg, 223Tyr, 231Ile, 242His, 265Asp, 301Ser, 330Gln, 334Asp | -15.3 | 1206.9 |
| Segment 5 | 1Met, 5Ser, 197Tyr, 208Gln, 210Asp, 235Ser, 237Lys | -19.7 | 1279.3 |
| Segment 6 | 1Met, 26Phe, 224Gln, 226Trp, 244Ser, 292Arg, 302Ser, 306Thr | -14.2 | 1264.5 |
| Segment 7 | 4Lys, 94Gln, 138His, 140Thr, 141Lys, 149Thr, 150Arg, 157Arg, 176Trp, 178Ser, 181Arg | -2.5 | 1073.9 |
| Segment 8 | 41Val, 42Gly, 44Asp, 47Ser, 49Thr, 60Phe, 66Lys, 74Arg, 78Arg, 84Arg | 0.7 | 927.3 |
| Segment 9 | 2Val, 4Ser, 74Thr, 84Gln, 108Arg, 116Arg, 118Leu | -9.2 | 860.7 |
| Segment 10 | 53Glu, 58Trp, 60Asp, 61Glu, 63Asp, 64Leu, 65Glu, 86Arg, 91Asp | -5.3 | 1035.0 |

Table S2. The frequencies of mutation types under different mutation energy thresholds.

| Mutation energy < -2 kcal/mol | | | Mutation energy < -1.5 kcal/mol | | |
| --- | --- | --- | --- | --- | --- |
| Amino acid site | Mutation type | Frequency | Amino acid site | Mutation type | Frequency |
| **404** | **Ser>Tyr** | **7** | 404 | Ser>Trp | 8 |
| 404 | Ser>Trp | 6 | **404** | **Ser>Tyr** | **7** |
| **395** | **Thr>Phe** | **5** | 407 | Ala>Tyr | 7 |
| 404 | Ser>Phe | 5 | **390** | **Ser>Phe** | **6** |
| **390** | **Ser>Phe** | **4** | **395** | **Thr>Phe** | **6** |
| 391 | Ser>Tyr | 4 | 390 | Ser>Tyr | 6 |
| 395 | Thr>Trp | 4 | 390 | Ser>Trp | 6 |
| 385 | Gly>Arg | 3 | 395 | Thr>Trp | 6 |
| 390 | Ser>Trp | 3 | 404 | Ser>Phe | 6 |
| 390 | Ser>Tyr | 3 | 404 | Ser>Trp | 6 |

Table S3. Primers for detection of immune-related gene expression.

| genes | Accession no. |  | Sequences |
| --- | --- | --- | --- |
| *β-actin* | KJ126772.1 | Forward | GGCATCACACCTTCTACAACGA |
|  |  | Reverse | ACGCTCTGTCAGGATCTTCA |
| *IgM* | KC677037.1 | Forward | CCACGAAGATTTGATTGAGCC |
|  |  | Reverse | TTCCTCGTCATCCACAAGCC |
| *IL1β* | XM_019365841 | Forward | CAGTGAAGACCGCAAAGTGC |
|  |  | Reverse | TATCCGTCACCTCCTCCAG |
| *IL8* | XM_019359413.2 | Forward | GCACTGCCGCTGCATTAAG |
|  |  | Reverse | GCAGTGGGAGTTGGGAAGAA |
| *CD4* | XM_005455473.3 | Forward | AAGAAACAGATGCGGGAGAGT |
|  |  | Reverse | AGCAGAGGGAACGACAGAGAC |
| *CD8* | XM_005450353.3 | Forward | GCTGGTAGCTCTGGCCTTT |
|  |  | Reverse | TGTGATGGTGTGGGCATCTC |
| *TNF-α* | AY428948 | Forward | GAACACTGGCGACAAAACAGA |
|  |  | Reverse | TTGAGTCGCTGCCTTCTAGA |
| *INF-γ* | KF294754.1 | Forward | CCAACAACTCAGGCTCGCTA |
|  |  | Reverse | TGCTCATGGTAGCGGTGTTT |
| *MHC-Iα* | XM_019355579.2 | Forward | TTCTCACCAACAATGACGGG |
|  |  | Reverse | AGGGATGATCAGGGAGAAGG |
| *MHC-II* | JN967618.1 | Forward | AGTGTGGGGAAGTTTGTTGGAT |
|  |  | Reverse | ATGGTGACTGGAGAGAGGCG |
| *IL2* | XM_025907658.1 | Forward | GTCCACCCATACCAGACCCA |
|  |  | Reverse | GCATCCACAGGCGAGGTTA |
| *IL4* | XM_025898051.1 | Forward | GGAGGGCAATCTGAGTCGT |
|  |  | Reverse | TGGGGAGGTGTTCAAAGC |

Table S4. PCR primers used in this study.

| Primer | Sequence (5'-3') | Purpose |
| --- | --- | --- |
| VH-F1 | TCAGGCCCAGCCGGCCATGGTTAAAAGRCCTGGA | Construction of scFv library |
| VH-F2 | TCAGGCCCAGCCGGCCATGACCATCACCTGTCAGGTC |  |
| VH-F3 | TCAGGCCCAGCCGGCCATGTCTCACAGATTGACCTGT |  |
| VH-R | *GCCACCGCCAGAGCCACCTCCGCCTGAACCGCCTCCACC*  TGTGCCTTTTCCCCAGTA |  |
| VL-F1 | *GGCGGTTCAGGCGGAGGTGGCTCTGGCGGTGGCGGATCG*  ACTGTGACTCAGCCTCCAG |  |
| VL-F2 | *GGCGGTTCAGGCGGAGGTGGCTCTGGCGGTGGCGGATCG*  GTGACCATCAGCGCCACAG |  |
| VL-R1 | ATAAGAATGCGGCCGCTTTGGTTCCTCCACCAAA |  |
| VL-R2 | ATAAGAATGCGGCCGCACATCCAGTCTGGTTCCT |  |
| scFv1-F | CGCGGATCCATGTCTCACAGATTGACCTGT | Expression |
| scFv3-F | CGCGGATCCATGGTTAAAAGRCCTGGA |  |
| scFv-R | CCCAAGCTTTTTGGTTCCTCCACCAAA |  |
| TiLV-112-F | CTGAGCTAAAGAGGCAATATGGATT | Detection |
| TiLV-112-R | CGTGCGTACTCGTTCAGTATAAGTTCT |  |
